# Green synthesis of silver nanoparticles and its environmental sensor ability to some heavy metals

**DOI:** 10.1186/s13065-023-01105-y

**Published:** 2024-01-06

**Authors:** Nesma H. Ibrahim, Gharib M. Taha, Noura Sh. A. Hagaggi, Marwa A. Moghazy

**Affiliations:** 1 Environmental Applications of Nanomaterial’s Lab., Department of Chemistry, Faculty of Science, Aswan University, Aswan, 81528 Egypt; 2 Botany Department, Faculty of Science, Aswan University, Aswan, 81528 Egypt

**Keywords:** Acacia raddiana, AgNPs, Colorimetric sensor, Hg^2+^, Cu^2+^, Pb^2+^, Co^2+^

## Abstract

This study marks a pioneering effort in utilizing *Vachellia tortilis* subsp. raddiana (Savi) Kyal. & Boatwr., (commonly known as *acacia raddiana*) leaves as both a reducing and stabilizing agent in the green “eco-friendly” synthesis of silver nanoparticles (AgNPs). The research aimed to optimize the AgNPs synthesis process by investigating the influence of pH, temperature, extract volume, and contact time on both the reaction rate and the resulting AgNPs' morphology as well as discuss the potential of AgNPs in detecting some heavy metals. Various characterization methods, such as UV–vis spectroscopy, X-ray diffraction (XRD), scanning electron microscopy (SEM), infrared spectroscopy (IR), Zeta sizer, EDAX, and transmitting electron microscopy (TEM), were used to thoroughly analyze the properties of the synthesized AgNPs. The XRD results verified the successful production of AgNPs with a crystallite size between 20 to 30 nm. SEM and TEM analyses revealed that the AgNPs are primarily spherical and rod-shaped, with sizes ranging from 8 to 41 nm. Significantly, the synthesis rate of AgNPs was notably higher in basic conditions (pH 10) at 70 °C. These results underscore the effectiveness of acacia raddiana as a source for sustainable AgNPs synthesis. The study also examined the AgNPs' ability to detect various heavy metal ions colorimetrically, including Hg^2+^, Cu^2+^, Pb^2+^, and Co^2+^. UV–Vis spectroscopy proved useful for this purpose. The color of AgNPs shifts from brownish-yellow to pale yellow, colorless, pale red, and reddish yellow when detecting Cu^2+^, Hg^2+^, Co^2+^, and Pb^2+^ ions, respectively. This change results in an alteration of the AgNPs' absorbance band, vanishing with Hg^2+^ and shifting from 423 to 352 nm, 438 nm, and 429 nm for Cu^2+^, Co^2+^, and Pb^2+^ ions, respectively. The AgNPs showed high sensitivity, with detection limits of 1.322 × 10^–5^ M, 1.37 × 10^–7^ M, 1.63 × 10^–5^ M, and 1.34 × 10^–4^ M for Hg^2+^, Cu^2+^, Pb^2+^, and Co^2+^, respectively. This study highlights the potential of using acacia raddiana for the eco-friendly synthesis of AgNPs and their effectiveness as environmental sensors for heavy metals, showcasing strong capabilities in colorimetric detection**.**

## Introduction

The word nanotechnology refers to the synthesis of novel materials on a nanoscale (1 to 100 nm), compared to the material in its bulk form. They exhibited higher capacity and surface area (ratio of area to volume) [1, 2].

Nanotechnology finds application across multiple domains such as optics [3], chemical sectors [4], electronics [5], energy research [6], photocatalysis [7, 8], efficient drug delivery systems [9], sensor technology, solar energy harvesting, and fuel cells [10], along with environmental uses [11, 12]. It is also utilized in hydrogen storage mechanisms, supercapacitors [13], biomedical fields including in vitro antialzheimer's studies [14], and the food industry [15].

The scientific community is very interested in the synthesis of AgNPs because of their wide variety of applications [1, 16, 17]. AgNPs have demonstrated antibacterial efficacy against numerous infectious and harmful pathogens, including multidrug-resistant bacteria [2]. As a result of the improved antibacterial activity of Ag at the nanoscale, AgNPs are incorporated into numerous types of products, such as the medical and healthcare fields, clothing, cosmetics, dental products, catheters, dressings, as well as surgical and food-handling instruments [18, 19]. In addition, AgNPs are applied as potential sensors not only for heavy metals detection [20, 21] but also for various ecological sensing purposes. This is because AgNPs are simple, adaptable, and inexpensive materials [22].

A wide range of physical and chemical methods are employed in the production of nanoparticles (NPs). For the synthesis of silver nanoparticles (AgNPs), physical methods like lithography, irradiation, laser ablation, evaporation, and condensation are commonly used [23–25]. These techniques might utilize thermal energy (physical vapor deposition), mechanical energy (ball milling) [26], electrical energy (electrical arc discharge), or light energy (laser ablation) [27]. Physical methods are advantageous due to the reduced toxicity of the reducing and stabilizing agents used, and they also produce AgNPs with small crystallite sizes and high purity. However, the downsides include substantial energy consumption and potentially low yield rates [28, 29]. In contrast, several chemical techniques are also utilized for AgNPs synthesis, including chemical reduction [30], the sol–gel method [31], microemulsion techniques [32], photochemical methods [33], electrochemical synthesis, and microwave-assisted synthesis [34]. While these chemical approaches may lead to high production costs and potentially hazardous by-products, they are capable of producing NPs that are free from aggregation and have a high yield [35, 36].

**Fig. 1:**
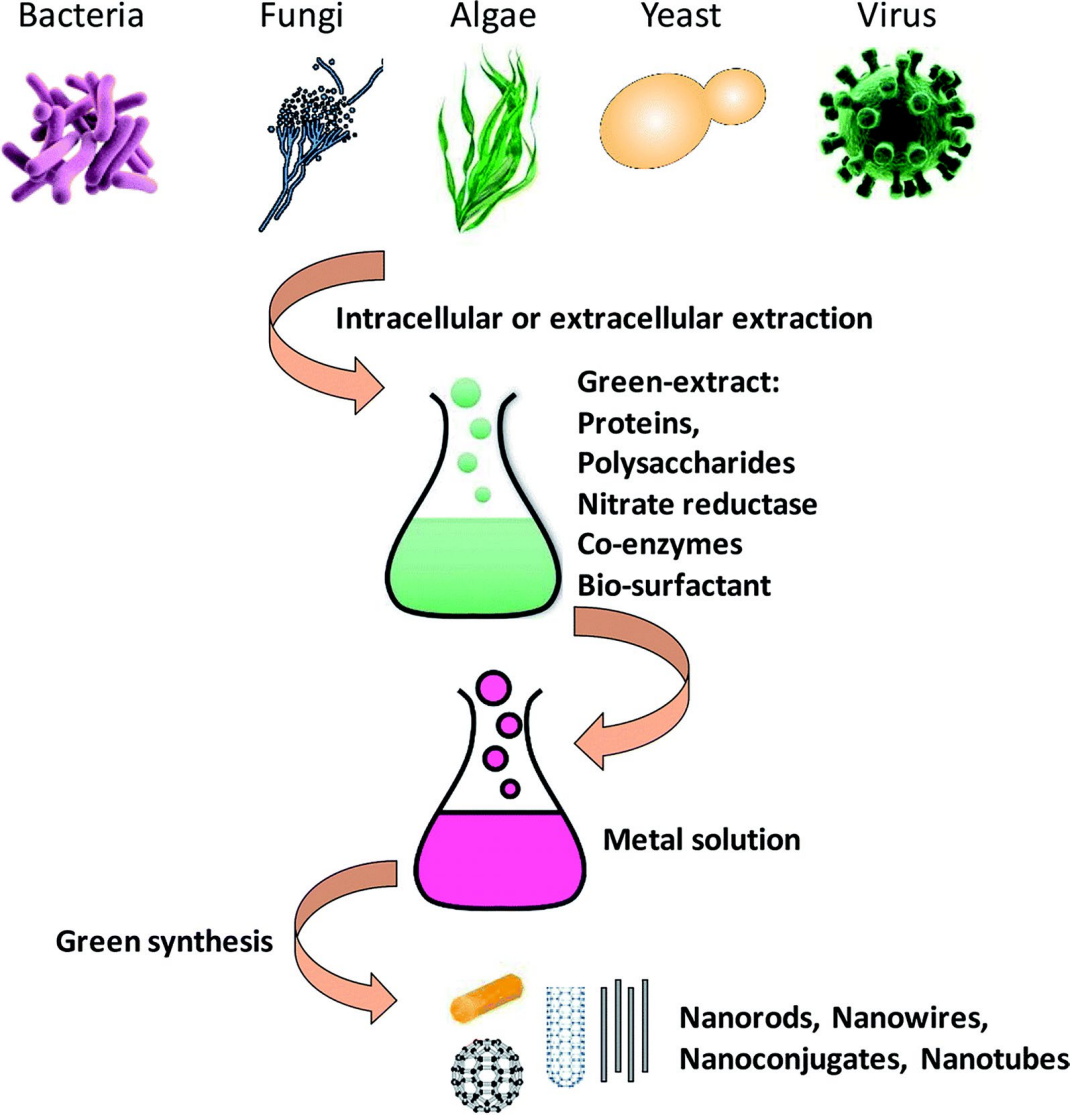
Biological resources work as reducing and stabilizing agents in the metal nanomaterials synthesis

Biological syntheses are favored today because they are secure, hygienic, affordable, simple to scale up and eco-friendly [37]. It does not need external reducing, capping, and stabilizing agents, so that, it does not require harmful or dangerous chemicals [38, 39]. The biological manufacturing of nanoparticles incorporates the adoption of multicellular and unicellular biological entities such as bacteria, actinomycetes, fungi, plants, viruses and yeasts [40, 41]. Recent studies have shown that the bio-synthesis of NPs using extracts from plants is an appealing substitute for conventional chemical synthesis and the more difficult microbe culturing and isolation procedures [42, 43], where the combinations of compounds contained in plant extract behave as reducing and stabilizing agents in the synthesis process. Although the complexity of their chemical composition, biological molecules have the advantage of being eco-friendly [44]. In plant extracts, bioactive alkaloids, phenolic acids, polyphenols, proteins, carbohydrates, and terpenoids are expected to be important in reducing the metal ions and then stabilizing them Fig. 1 [45, 46].

*Vachellia tortilis *subsp.* raddiana (Savi) Kyal. & Boatwr. (Syn: Acacia tortilis subsp. raddiana (Savi) Brenan) (acacia raddiana)* grows as wild flora in the Siwa Oasis and Aswan (Elephantine Island). By the end of the twenty-first century, Egypt's oases will have an entirely different plant life thanks to the Toschka canal, which will redirect the Nile River from Lake Nasser (south of Aswan on the Nile Valley) to the Kharga Oasis and then the Farafra Oasis in the Western Desert. This project is expected to reclaim and cultivate about 500,000 acres [47]. The *acacia raddiana* plant offers benefits such as year-round availability and cost-effectiveness. Moreover, its leaf extract is rich in phenolic compounds, including flavonoids, tannins, alkaloids, and saponins [48].

**Fig. 2:**
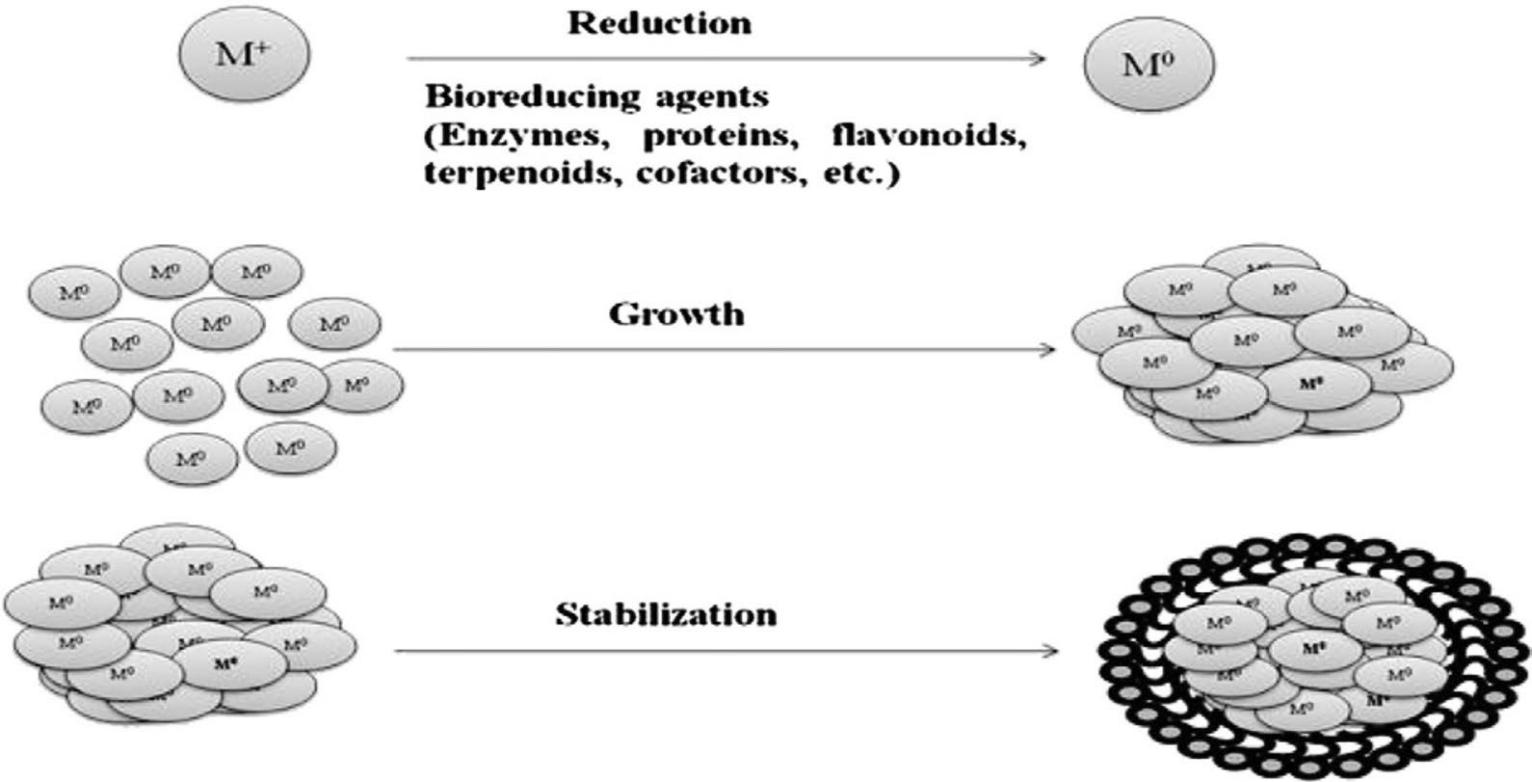
Mechanism of AgNPs synthesis using plant extract

In the preparation of nanoparticles utilizing plant extracts, the extracts are simply added to a metal salt solution at the optimal temperature. Within minutes, the reaction was completed. AgNPs and numerous other metals have been produced via this mechanism Fig. 2 [49].

This research concentrated on detecting heavy metals using the synthesized AgNPs as sensors, owing to the toxic and detrimental effects these elements can have on the environment. The elements investigated include the following…

Mercury is a heavy metal that may be harmful to people's health and is a hazardous element in the environment. Mercury was ranked among the top ten substances or chemical groups that the World Health Organization assessed to be of the greatest public health concern [50, 51]. Due to the presence of mercury in water and soil samples in large quantities, it accumulates in the ecological food chain. As a result, both the human population and other living organisms are negatively impacted [52]. There are several sources of mercury as cinnabar rocks, which are formed through volcanic activity or the natural weathering of rock. It has up to 86% of its content mercury which releases mercury in various forms to the environment [53, 54]. As well, human activity through coal combustion, electrical power production and industrial waste disposal is the main source of mercury contamination in the environment [45]. Also, the biologically harmful form of methyl mercury (MeHg) which accumulates in the tissue of fish and birds and is released to the environment by microorganisms that live in soil and water [55].

An essential mineral that is available in dietary supplements and is a part of a balanced diet is copper (II) ion. However, excessive Cu^2+^ ion concentrations in humans can be hazardous and cause serious illnesses like dyslexia and hypoglycemia [56]. Numerous industries generate Cu^2+^ ions throughout their production methods, which can get into the food chain and build up in people [57, 58].

Lead (Pb) is the most prevalent harmful heavy metal in the environment, that was found in our atmosphere [59]. Additionally, it is considered one of the five heavy metals that are commonly found in water, along with Hg, As, Cr, and Cd [60, 61]. Lead is still utilized in the creation of paint, and car batteries as well as in recovering lead metal electrodes [62, 63]. Therefore, the rate of paint deterioration and recycling of automotive batteries leads to increasing the amount of lead in both soil and water in particular places. Since lead is a hazardous metal, aquatic life is constantly at risk from its presence in the environment. High blood levels of Pb^2+^ can seriously harm a human's health, especially in young kids. Children's blood serum contains abnormally high levels of Pb^2+^ in a certain region which exceeds the recommended value of 1.00 µg/L [64, 65]. Consequently, accurate Pb^2+^ determination in biological and environmental materials is crucial.

High quantities of cobalt in the human body lead to cobalt toxicity, which has catastrophic consequences. Cobalt and its salts are found in a variety of products, including enamels, porcelain, acrylic paints on glass, grinding wheels, semiconductors, electroplating, hygrometers, and nuclear therapies [66]. Asthma, heart, thyroid, and liver issues are some of the negative symptoms of cobalt toxicity [67]. Moreover, it irritates the eyes and mucous membranes, making breathing through the nose extremely uncomfortable and frequently resulting in perforation of the nasal septum [68].

The mercury, copper, lead and cobalt contents of various samples have been investigated via a variety of methods including cold vapor atomic absorption [69]**,** cold vapor atomic fluorescence spectroscopy [70], atomic absorption spectrometry [71], inductively coupled plasma atomic emission spectrometry [72], electrothermal atomic absorption spectrometry, atomic fluorescence spectrometry [73], fluorescent probes [74], inductively coupled plasma-mass spectrometry [75] and electrochemical detection [76]. Among them, the development of sensors to measure, monitor, and detect heavy metals in environmental samples is a crucial part of today's technology.

Numerous studies are concentrating on the fascinating field of creating hydrophilic noble metal nanoparticles capable of binding and coordinating with heavy metal ions. Among various noble metals, silver nanoparticles stand out as the most appropriate candidates for plasmonic analyte detection [77, 78]. The type of capping agent used significantly affects the nanomaterial's hydrophilic or hydrophobic properties, as well as its tendency to aggregate or self-assemble [79]. Additionally, the presence of specific functional groups can enhance the particles' interactions with nearby substances, thereby increasing their selectivity and sensitivity towards these substances [80]. Surface functionalization is a crucial strategy for enhancing selectivity and sensitivity in the detection and removal of pollutants in water contaminated with heavy metals [81].

So, using AgNPs, colourimetric sensing of cobalt, copper, lead, and mercury is an efficient, fast, ecologically safe, and highly active technique [82].

This study presents a simple, quick, and eco-friendly method for synthesizing silver nanoparticles (AgNPs) using aqueous extracts of *acacia raddiana* leaves. It focuses on examining various parameters in the biosynthesis process to establish optimal synthesis conditions. The study also investigates the potential of the synthesized AgNPs as colorimetric sensors for detecting cobalt, copper, lead, and mercury ions. This study stands apart from previous research in several ways. Notably, acacia raddiana has not been previously employed in nanoparticle synthesis. The physical and chemical properties (such as size and shape) of the nanoparticles synthesized in this study are in line with standard benchmarks. Additionally, this study achieves a quicker response time for Cu^2+^, Hg^2+^, Co^2+^, and Pb^2+^ ions compared to earlier studies. Furthermore, the selection of the study area aligns with Egypt's Vision 2030, specifically under the “Environmental Sustainability” section, which focuses on “the sustainable and integrative environment.” This is reflected in the study's goal to treat water contaminated with heavy metals using simple methods, aiming to conserve water resources amidst global water scarcity issues.

## Materials and methods

### Materials

All used chemicals were of analytical grade. Silver nitrate (AgNO_3_, PRATAP, UFC, PVT. LTD, India), concentrated nitric acid (HNO_3_ 65%, DOP, Torkiye), Ammonium hydroxide (NH_4_OH 30%, DOP, Torkiye), copper sulphate pentahydrate (Cu SO_4_. 5H_2_O, DOP, Torkiye), mercuric sulphate (HgSO_4_, S. d. FiNE-CHEM Ltd, India), nickel chloride hexahydrate (Ni Cl_2_. 6H_2_O, Oxford Lab. Reagent, India), cobalt nitrate hexahydrate (Co (NO_3_)_2_. 6H_2_O, Loba Chemie PVT LTD, India), chromium nitrate nonahydrate (Cr (NO_3_)_3_. 9H_2_O, Loba Chemie PVT LTD, India), cadmium nitrate tetrahydrate (Cd (NO_3_)_2_. 4H_2_O, RIEDEL–DE HAEN AG SEELZE-HANNOVER) and lead nitrate (Pb (NO_3_)_2_, UFC, PVT. LTD, India).

### Procedure

#### Plant extract preparation

*Vachellia tortilis *subsp.* raddiana (Savi) Kyal. & Boatwr. (Syn: Acacia tortilis subsp. raddiana (Savi) Brenan) (acacia raddiana)* leaves were obtained from Elephantine Island in Aswan Government, Egypt, specified by Prof. Mohamed Gabr Sheded, Professor of Plant Ecology & Flora, Botany Department, Faculty of Science, Aswan University.

The plant leave is available in Aswan University Herbarium (ASW. Herbarium) with NO. 11822. The samples were collected with the permission of Aswan University which is considered the main research body in the Aswan government.

**Fig. 3:**
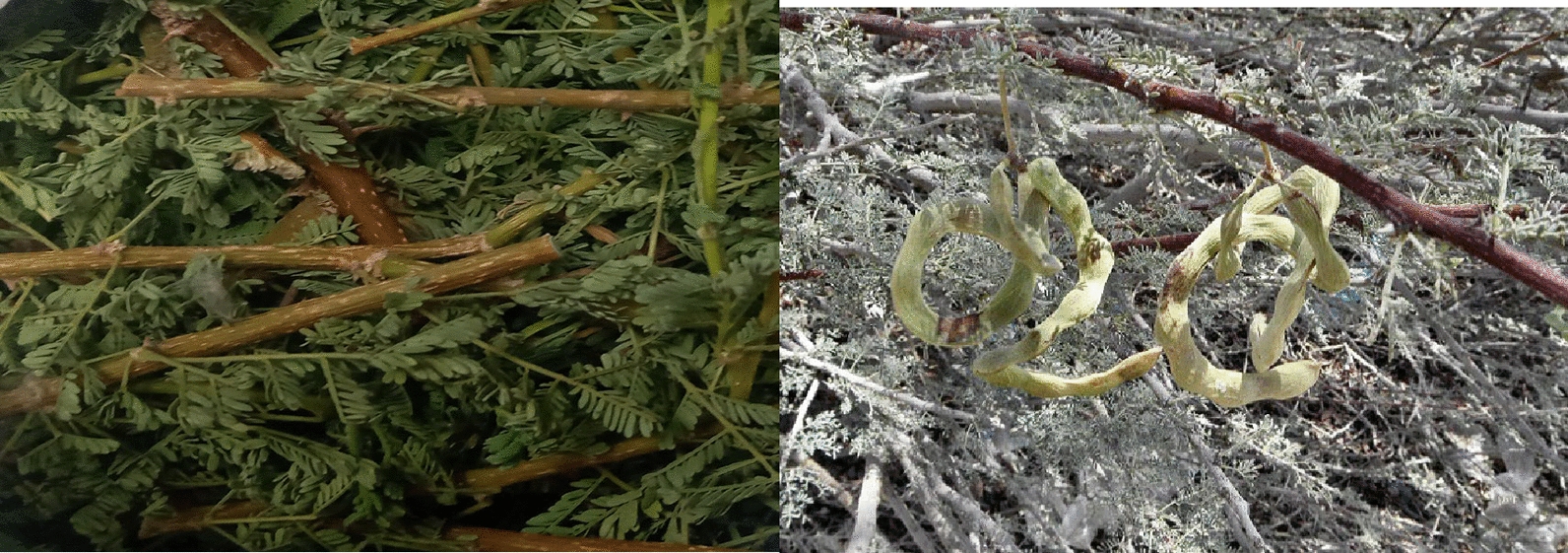
Acacia raddiana plant

Ten g of acacia raddiana leaves, as shown in Fig. 3, were initially rinsed with distilled water to remove impurities. The leaves were air-dried for two days and then ground into a powder. This powder was then combined with 100 mL of distilled water and boiled for 25 min. After allowing the mixture to cool at room temperature, it was filtered using Whatman filter paper no. 41. The filtrate obtained was used as the plant extract.

#### Phytochemical analysis of acacia raddiana leaf extract

The acacia raddiana leaf extract was subjected to various standard tests to detect different phytochemicals [83–85]. The methods used for these tests are described as follows:

- Tannins: A mixture of 50 mg of the extract in 5 mL of distilled water was heated in a water bath and then filtered. The addition of ferric chloride to the filtrate until it turned dark green confirmed the presence of tannins.
- Saponins: 0.2 g of the plant extract was boiled in 5 mL of distilled water. The formation of persistent foam was an indication of saponins.
- Steroids: 20 mg of the extract was mixed with 1 mL of methanol, filtered, and then treated with 1 mL of concentrated H_2_SO_4_. A yellow-green fluorescence signified the presence of steroids.
- Terpenoids: 0.5 g of the extract was combined with 2 mL of chloroform, followed by the gradual addition of concentrated H_2_SO_4_. A reddish-brown color at the interface indicated terpenoids.
- Flavonoids: 0.2 g of plant extract was dissolved in diluted NaOH, changing the solution from yellow to colorless upon gradual addition of HCl, a sign of flavonoids.
- Anthraquinone: 0.5 g of the extract was mixed with 5 mL of chloroform, shaken for 5 min, and filtered. Adding a 10% ammonia solution to the filtrate, and observing a change to pink, violet, or red in the ammonia layer indicated anthraquinone.
- Phenolic compounds: To 50 mg of the extract, 3 mL of a 10% lead acetate solution was added. The large white precipitate formed suggests that phenolic chemicals are present.

#### Synthesis of AgNPs

**Fig. 4:**
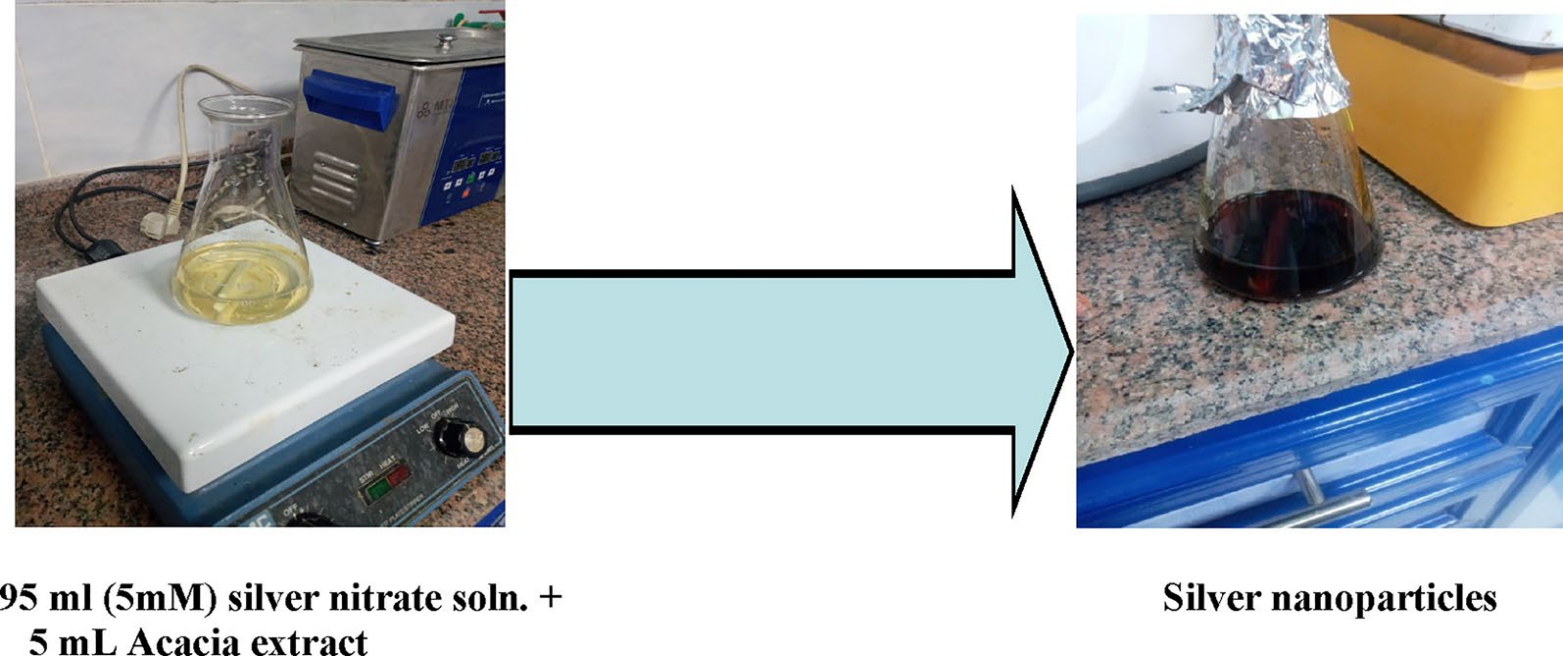
Acacia extract and silver nanoparticles formation

Five mL of acacia raddiana leave extract was mixed with 95 mL of a (5 mM) AgNO_3_ solution, and the obtained mixture was stirred at 70 °C for 2 h. As shown in Fig. 4, the colour of the solution changes from pale yellow to reddish brown. The resulting precipitate was separated by adding ethanol and decantation the clear solution, then dried at 50–80 °C.

##### Effect of pH

In this part of the study, 5 mL of acacia raddiana leaf extract and 95 mL of a 5 mM AgNO_3_ solution were mixed, and their pH was adjusted to a range between 4 and 10 using 1 M HCl and 1 M NaOH. The resulting mixture was then stirred at a temperature of 70 °C for 2 h.

##### Effect of temperature

Here, the temperature of the mixture, consisting of 5 mL of acacia raddiana leaf extract and 95 mL of a 5 mM AgNO_3_ solution, was modified to 50, 70, and 90 °C. This was done at the previously determined optimal pH, and the mixture was stirred for 2 h.

##### Effect of extract volume

The volume of the extract was varied from 1 to 10 mL, which was then combined with 95 mL of a 5 mM AgNO_3_ solution at the optimum pH and temperature. Following this, the mixture was stirred for 2 h.

##### Effect of stirring time

For this aspect, the synthesis of AgNPs was examined over stirring times of 0.5, 1, 2, 3, or 4 h. This was conducted using 95 mL of a 5 mM AgNO_3_ solution, maintaining the optimal pH, temperature, and extract volume settings.

#### Characterization techniques

The synthesized AgNPs were analyzed using a Bruker AXS D8 X-ray diffraction (XRD) system from Germany, employing Cu Kα radiation at a wavelength of 0.154 nm. Field Emission-Scanning Electron Microscopy (FE-SEM, QUANTAFEG250, The Netherlands) was utilized at a voltage of 20 kV. Additionally, a JEOL (JEM-HR-2100 ELECTRON MICROSCOPE, USA) Transmittance Electron Microscope (TEM) was used for further examination. Fourier Transform-Infrared (FTIR) analysis was conducted using a JASCO 3600 (Tokyo, Japan), assisted by Agilent Technologies' Cary 630, to measure spectral transmittance at room temperature. These spectral measurements covered a range from 400–4000 cm^−1^ with a spectral resolution of 2 cm^−1^, aiming to identify and analyze the functional groups present in the plant extract. The particle size distribution and charge characteristics of the AgNPs were assessed using a Zeta sizer Ver. 7.03 (temperature 25 °C, count rate 293.2 kcps, measurement position 2 mm). Furthermore, an EDAX APEX and a UV-1800 TOMOS spectrophotometer from China were employed at room temperature to determine the maximum wavelength (λ max.) of the synthesized AgNPs.

#### Study of sensing activity

Six different metal salts were used to test the green synthesized AgN Ps' ability to detect metal ions, i.e. [Cu SO_4_. 5H_2_O, HgSO_4_, Ni Cl_2_.6H_2_O, Co (NO_3_)_2_. 6H_2_O, Cr (NO_3_)_3_. 9H_2_O, Pb (NO_3_)_2_ and Cd (NO_3_)_2_. 4H_2_O] were dissolved in distilled water to get a standard solution (0.1 M) for every salt for the colorimetric investigation. To a 1 mL light brown suspension of AgNPs (9.2 × 10^–4^ M), 2 mL of each metal salt solution (0.001 M) was added. The effect of pH was investigated at pH 2, 7 and 10 using a solution of 1 M NaOH or HNO_3_. At the optimum conditions of pH, the influence of dose was investigated in the range of 25 to 500 ppm, the resulting suspensions were analyzed by spectrophotometry from 200—1000 nm. The concentration effect of metal salt solution was also studied from 1 × 10^–7^ to 1 × 10^–2^ M. Additionally, the effect of time was studied for each concentration from 0 to 15 min.

#### Real sample analysis

The synthesized AgNPs were applied to analyze a real wastewater sample. This sample was sourced from the Egyptian Company for Chemical Industries-KIMA, located in Aswan Governorate, Upper Egypt. The water was collected from the effluent pit (82 BS211) unit and then filtered through double-ring filter paper no. 102 to remove suspended particles before analysis. The detection of each metal was conducted using the AAS technique under optimal conditions.

## Results and discussion

### Phytochemical analysis of leaf extract

Table 1 presents the findings from the analysis of phytochemical constituents in the leaf extract. The study revealed a high presence of saponins, alkaloids, and phenolic compounds, along with moderate levels of tannins and flavonoids; however, anthraquinone and terpenoids were absent. These compounds are essential for converting silver ions into silver nanoparticles through various functional groups like hydroxyl, ketone, and aldehydes [86, 87]. They also serve as stabilizing agents.

**Table 1:** Phytochemical analysis of acacia raddiana leaf extract

Phytochemical examined	Results
Phenolic compounds	++
anthraquinone	−
Flavonoids	+
Tannins	+
Steroids	−
Saponins	++
Alkaloids	++
Terpenoids	−

(−) absent/(+) moderate presence/(++) abundant presence

### Characterization of AgNPs

#### UV–Vis spectroscopy

**Fig. 5:**
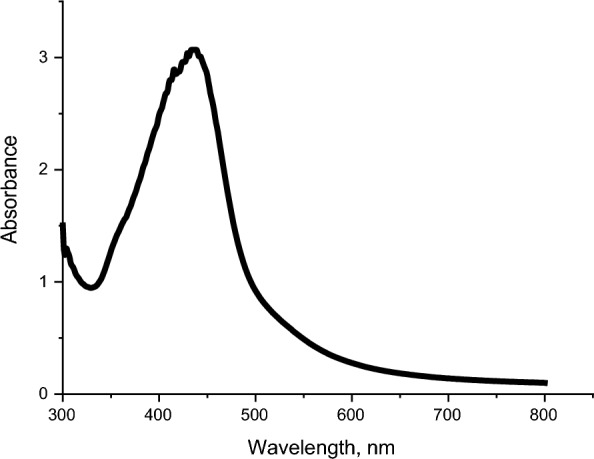
UV–visible spectroscopy for silver nanoparticles synthesized using 95 mL (5 mM) of AgNO_3_ mixed with 5 mL of acacia extract at pH10 and stirring time 2 h

AgNPs exhibit a surface plasmon resonance (SPR) peak at 400–500 nm, which may be utilized to confirm the formation of AgNPs using UV–Vis spectroscopy [88]. According to Fig. 5, the maximum absorbance peak in the absorption spectra of AgNPs is located at 423 nm.

**Fig. 6:**
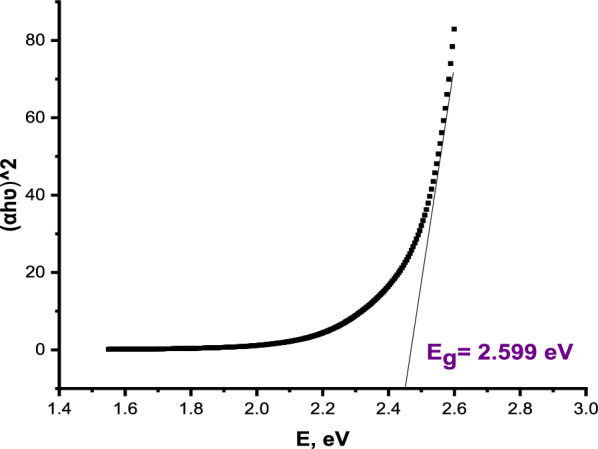
Tauc’s plot of the absorption spectra of AgNPs

The band gap of AgNPs can be estimated using the absorption spectra obtained from the UV–Vis spectrophotometer of the AgNPs suspended solution. The onset wavelength of absorption was determined by extrapolation of the baseline and the absorption edge as shown in Fig. 6. The band gap was calculated by Eq. (1) [89]:

1 $${\text{E}}=\frac{hc}{\lambda }$$

where h is Planck^’^s constant (6.626 × 10^–34^ Js), c is the speed of light (2.998 × 10^8^ m/s) and $\lambda$ is the cutoff wavelength of light × 10^–9^ m (510 nm). There is a conversion factor that should be used in calculations (Joule to eV where 1 eV = 1.63 × 10^–19^).

Another method for estimating the band gap is Tauc's equation (Eq. 2) [90] as follows:

2 $${\left(h\upsilon \alpha \right)}^{n}=A(h\upsilon -{E}_{g})$$

Here, depending on the nature of the transition, the plank constant, light frequency, absorption coefficient, proportional constant, energy gap, and power index are each represented by h, ʋ, α, A, E_g_, respectively.

Tauc Eq. (2) converts to Eq. (3) as follows:

3 $$h\upsilon =\frac{hc}{\lambda }=\frac{1240}{\lambda }$$

By substituting h and c with their values, the equation becomes:

4 $${\left(\alpha \frac{1240}{\lambda }\right)}^{n}=(\frac{1240}{\lambda }-Eg)$$

According to Eq. (4), the absorption value in the UV–vis spectrum is represented as (α), and the detection wavelength is λ. Plotting the (αhν)^2^ versus optical band gap energy, the E_g_ was estimated by extrapolating a straight line to the (αhν)^2^ = 0 axes [90–92]. Figure 6 illustrates that the band gap for AgNPs is 2.599 eV.

#### Zeta potential and size distribution

**Fig. 7:**
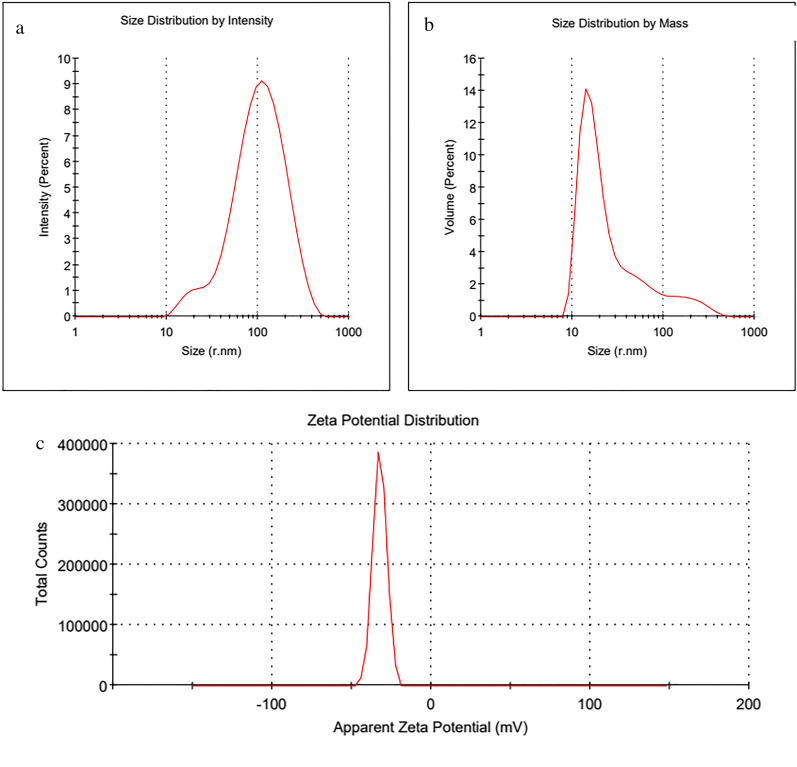
**a** Size distribution by intensity, **b** size distribution by mass of the synthesized AgNPs and **c** zeta potential of the synthesized AgNPs

The synthesized AgNPs' stability was evaluated using zeta potential measurements, with a higher positive or negative value suggesting greater nanoparticle stability [93]. The distribution of size, both by intensity and mass, is depicted in Fig. 7a, b, presenting a Z-average (± SD) of 77.35 ± 50.4 (r. nm). The AgNPs exhibited a negative zeta potential of − 32.2 mV, as shown in Fig. 7c, indicative of high stability. Prior studies suggest that a strong negative zeta potential enhances repulsion between nanoparticles, promoting their stable dispersion and contributing to their colloidal quality [93]. Furthermore, the stability of AgNPs influences their size, with reduced stability leading to particle aggregation and an increase in particle size [94].

#### FTIR spectroscopy for AgNO_3_, acacia raddiana leaves extract and AgNPs

**Fig. 8:**
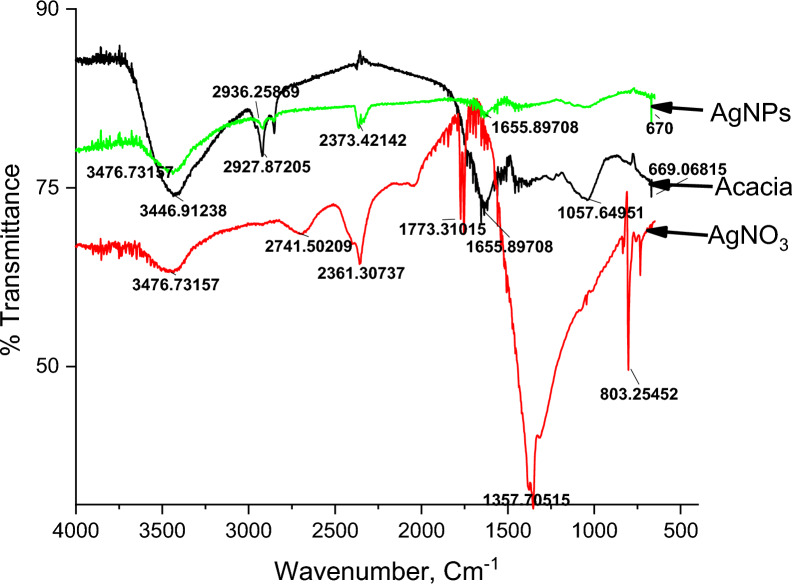
FTIR of acacia leave, silver nitrate and the synthesized silver nanoparticles

The functional groups or biomolecules that are responsible for the reduction of silver ions to AgNPs could be identified with the Fourier transform infrared (FTIR) spectroscopy. As depicted in Fig. 8. This can be achieved by comparing the intensity of AgNPs bands with standard AgNO_3_ and acacia values. The proportionate change in the band that was observed after silver nitrate treatment is probably indicating that the functional groups participated in the formation of AgNPs [95].

FTIR analysis for acacia leaves illustrates that four main peaks were observed at 1057, 1655, 2927 and 3446 cm^−1^. The first peak (1) is related to C–N amine and the peak at 1655 cm^−1^ for the carbonyl groups in amide linkages or stretching vibration of C=O group neighbor to carbon–carbon double bonds (C=C) [96]. C–H stretching appeared at 2927 cm^−1^ indicating the presence of alkanes [97] and the vibration band at 3446 cm^−1^ corresponding to the O–H bond possibly arising from water.

The AgNO_3_ sample analysis shows peaks as follows, 1357 cm^−1^ related to the N–O of the nitrate group and 3476 cm^−1^ corresponding to the O–H bond possibly arising from water [98].

Comparing AgNPs to Acacia extract and AgNO_3_, only four peaks formed. The peak at 1655 cm^−1^ is related to the carbonyl groups (C=O) which formed in low intensity compared with the same peak in acacia leaves which indicates the reaction takes place between AgNO_3_ and the extract. The second peak was observed at 2373 cm^−1^ which was attributed to the amino or amide groups and a stretching vibration band formed at 3476 cm^−1^ for O–H of water. Finally, a peak was observed at 670 cm^−1^ for Ag which confirms the AgNPs formation. The peaks at 1357 and 1773 cm^−1^ attributed to N–O and amino groups disappeared compared with the FTIR analysis of AgNO_3_ and AgNPs. The results indicate that a majority of the carbonyl, hydroxyl, amino, and amide groups present in the components of the plant extract attach to the surface of the synthesized AgNPs, functioning as capping agents to ensure stabilization. Based on these observations, it is noted that AgNPs demonstrate hydrophilic characteristics [99, 100].

#### X-ray diffraction analysis (XRD)

##### Effect of pH

**Fig. 9:**
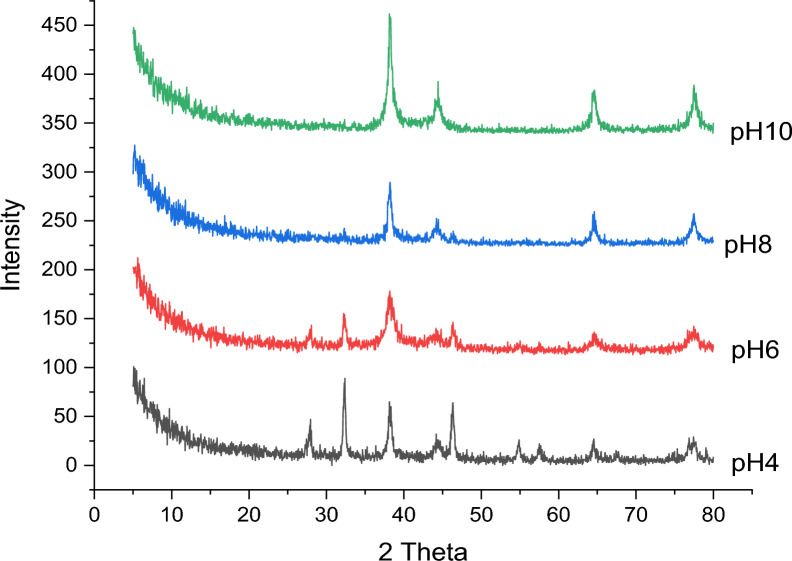
Effect of pH on AgNPs synthesis using 95 mL (5 mM) AgNO_3_ mixed with 5 mL of acacia raddiana extract at 70 °C and stirring time 2 h

XRD patterns can be used to investigate the nature or crystalline composition of biosynthesized AgNPs. XRD examination of AgNPs biosynthesized at varying pH levels (4–10) using a 5 mM AgNO_3_ solution mixed with 5 mL of the extract, stirred for 2 h at a temperature of 70 °C, is presented in Fig. 9.

The AgNPs synthesized at pH8 and pH10 show only four peaks at 2θ 38.18, 44.25, 64.72, and 77.40° corresponding to the AgNPs which agrees with the previous research [95, 101, 102]. The main peak at 2θ 38.18 for AgNPs prepared at pH10 is the highest intensity compared with other synthesized samples. Two peaks at 2θ 27.3 and 31.8° appeared for pH4 and pH6 samples may imply the presence of bio-organic molecules of the aqueous extract of the plant on the surface of AgNPs or due to the existence of Ag^+^ ion isn’t reduced [103, 104]. Therefore, the alkaline medium improved the ability of reduction and stabilizing agents in the leaf extract, on contrast, the acidic medium is unsuitable for AgNPs formation [105]. The synthesis of AgNPs under alkaline pH conditions offers several benefits, including enhanced stability, a higher yield of nanoparticles, faster growth, and an improved reduction process [106]. In plant extracts, the OH^−^ groups are crucial for their role as reducing and stabilizing agents in AgNPs synthesis. Therefore, a basic pH environment facilitates a greater participation of OH^−^ groups in the reduction reaction, which in turn improves the efficiency of the reduction process [107].

The crystallite sizes were determined by Scherer Eq. (5) to be 20.20, 10.05, 20.24 and 30.72 nm for pH4, pH6, pH8 and pH10, respectively.

5 $${\text{D}}=\frac{k\lambda }{\beta cos\theta }$$

where D is crystallite size, k is constant (0.89 < k < 1), λ is the wavelength of the X-ray source (0.1541 nm), β is the full width at half maximum (FWHM) and θ is the diffraction angle that corresponding to the lattice plane.

##### Effect of reaction temperature

The impact of temperature on the synthesis process was examined at 50, 70, and 90 °C, maintaining a pH of 10 and using a combination of 5 mM AgNO_3_ solution with 5 mL of extract, stirred for 2 h.

**Fig. 10:**
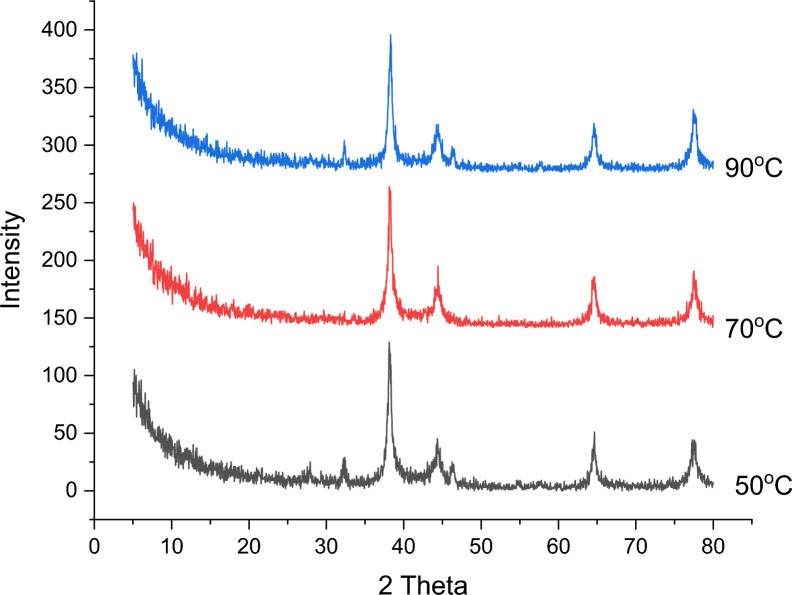
Effect of temperature on synthesis of AgNPs using 95 mL (5 mM) of AgNO_3_ mixed with 5 mL of acacia raddiana leave extract at pH10 and stirring time 2 h

As shown in Fig. 10, the sample synthesized at 70 °C shows a pure phase of AgNPs without any additional peaks of a second phase compared with 50 and 90 °C which exhibit a peak of second phase at 2θ = 32.2. The effect of temperature has little impact on the crystallite size of the produced samples. The crystallite sizes were calculated to be 23.70, 21.75 and 23.60 nm for 50, 70 and 90 °C, respectively. From these results, 70 °C is chosen as the optimum temperature because it gives the pure form of AgNPs with the smallest crystallite size. Previous research indicates that the ideal temperature for successful AgNPs synthesis falls between 60 and 80 °C [108].

##### Effect of extract volume

Due to the significant role that leaf extract plays in the Ag^+^ ion reduction, their volume up to a certain point is effective in the synthesis of AgNPs. For this study, varying volumes of leaf extract (1, 2.5, 5, 7.5, and 10 mL) were tested at pH 10 with a 5 mM AgNO_3_ solution, stirred for 2 h at 70 °C.

**Fig. 11:**
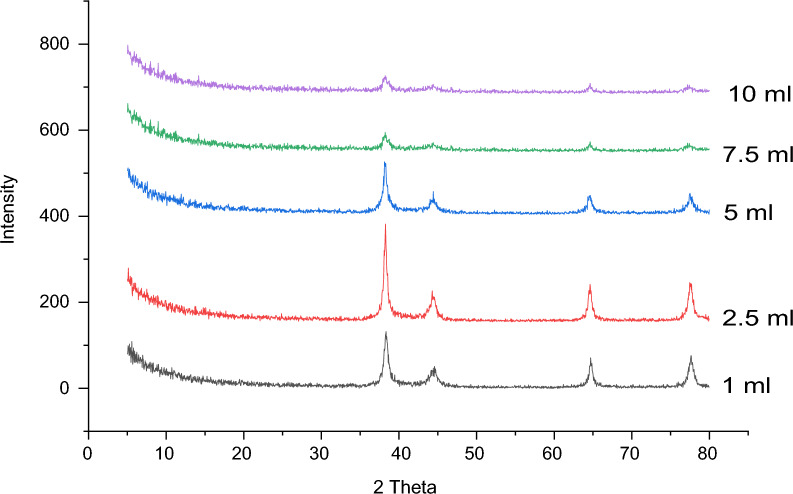
Effect of extract volume in synthesis of AgNPs Using 95 mL (5 mM) of AgNO_3_ at pH10, 70 °C and stirring time 2 h

As illustrated in Fig. 11, the intensity of the AgNPs peaks increases from 1 mL to be maximum at 2.5 mL then gradually decreases to be minimum at 10 mL.

The peak intensity increased from 1 to 2.5 mL because the number of active molecules in the acacia raddiana leaf extract responsible for AgNO_3_ reduction increased. In contrast, the decrease in intensity peaks above 2.5 mL is due to the presence of extract components that have not been used in the reduction process [109]. The optimal extract volume was determined to be 2.5 mL, aligning with past findings that suggest smaller volumes are more effective for nanoparticle synthesis [110].

##### Effect of stirring time

**Fig. 12:**
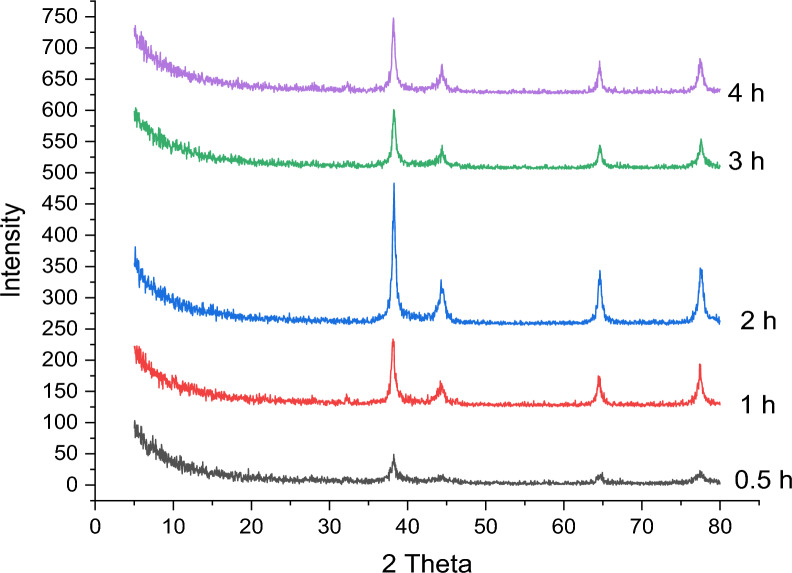
Effect of stirring time on the synthesis of silver nanoparticles using 95 mL (5 mM) of AgNO_3_ mixed with 2.5 mL of acacia raddiana at pH10, 70 °C

Additionally, the stirring time's effect was explored over a range from 0.5 to 4 h, using the same conditions of pH, AgNO_3_ concentration, extract volume, and temperature. The AgNPs synthesized at 2 h show the sharpest main peak at 2θ 38.21° compared with the other samples. The crystallite sizes were determined to be 20.24, 35.34, 35.35, 24.40 and 30.72 nm at 0.5, 1, 2, 3 and 4 h, respectively. Increasing the time from 0.5 to 2 h increases the main peak intensity of the synthesized AgNPs, which decreases gradually at 3 and 4 h as shown in Fig. 12.

#### AgNPs synthesis mechanism

**Fig. 13:**
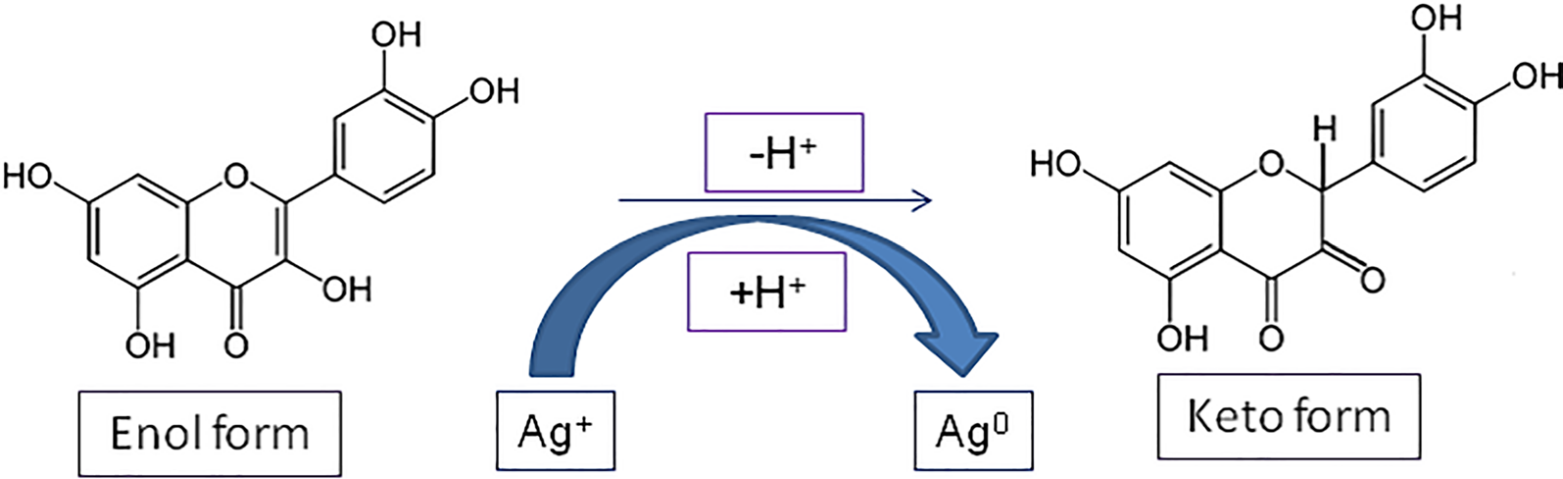
Mechanism of AgNPs synthesis

The synthesis of AgNPs is facilitated by the presence of various organic compounds in biological systems, capable of donating electrons for the conversion of Ag^+^ ions to Ag^0^. These compounds include carbohydrates, fats, proteins, enzymes, phenols, flavonoids, terpenoids, alkaloids, and others. The specific active components responsible for reducing silver ions vary based on the extract used. In hydrophytes, the dehydrogenation of acids (like ascorbic acid) and alcohols (such as catechol) plays a role. In mesophytes, transformations like keto to enol conversions (observed in compounds like cyperaquinone, dietchequinone, and remirin) are involved. Similarly, xerophytes plants may utilize either or both of these pathways to provide the necessary electrons for AgNPs transformation, as illustrated in Fig. 13 [111].

#### Scanning electron microscopy (SEM)

**Fig. 14:**
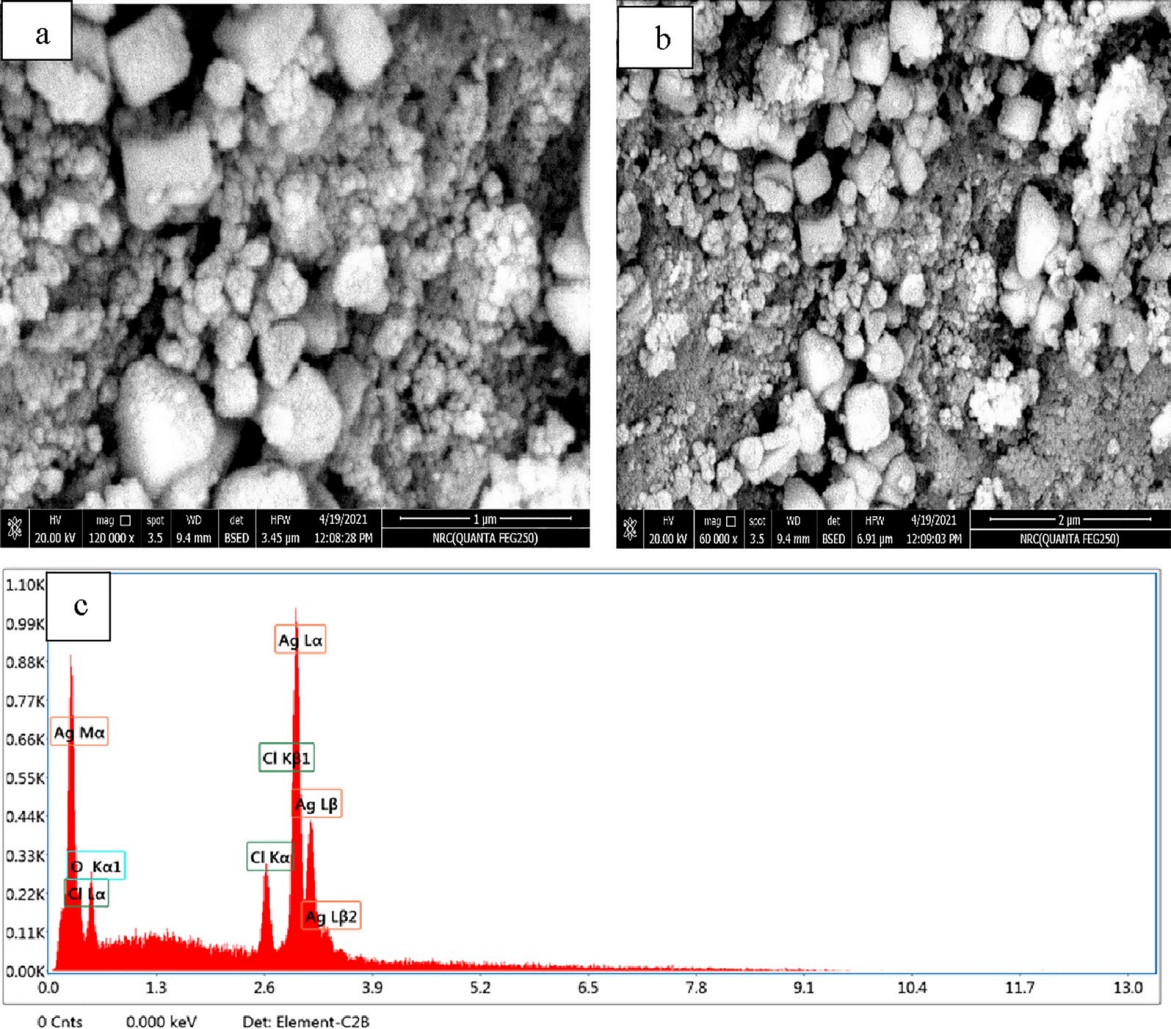
**a**, **b** SEM micrographs of synthesized AgNPs; **c** energy dispersive X-ray (EDX) spectrum of synthesized AgNPs

SEM Micrograph reveals the AgNPs morphology. The AgNPs synthesized at optimum conditions (pH10, 70 °C, 2.5 mL of acacia leaf extract and stirring time 2 h) were characterized by SEM micrograph as shown in Fig. 14a, b. It reveals that most of the synthesized nanoparticles are spherical with some irregular particles. Figure 14c displays the energy dispersive X-ray (EDX) spectrum, employed to assess the purity and composition of the green-synthesized AgNPs. The presence of AgNPs is confirmed by the prominent Ag peak in the EDX spectra at 3 keV [112]. Elemental analysis revealed a high silver content in the sample (73.86 wt%). Additionally, the absence of an N signal from AgNO_3_ suggests that acacia raddiana leaves effectively reduced Ag^+^ to Ag^0^.

#### Transmitting electron microscopy (TEM)

**Fig. 15:**
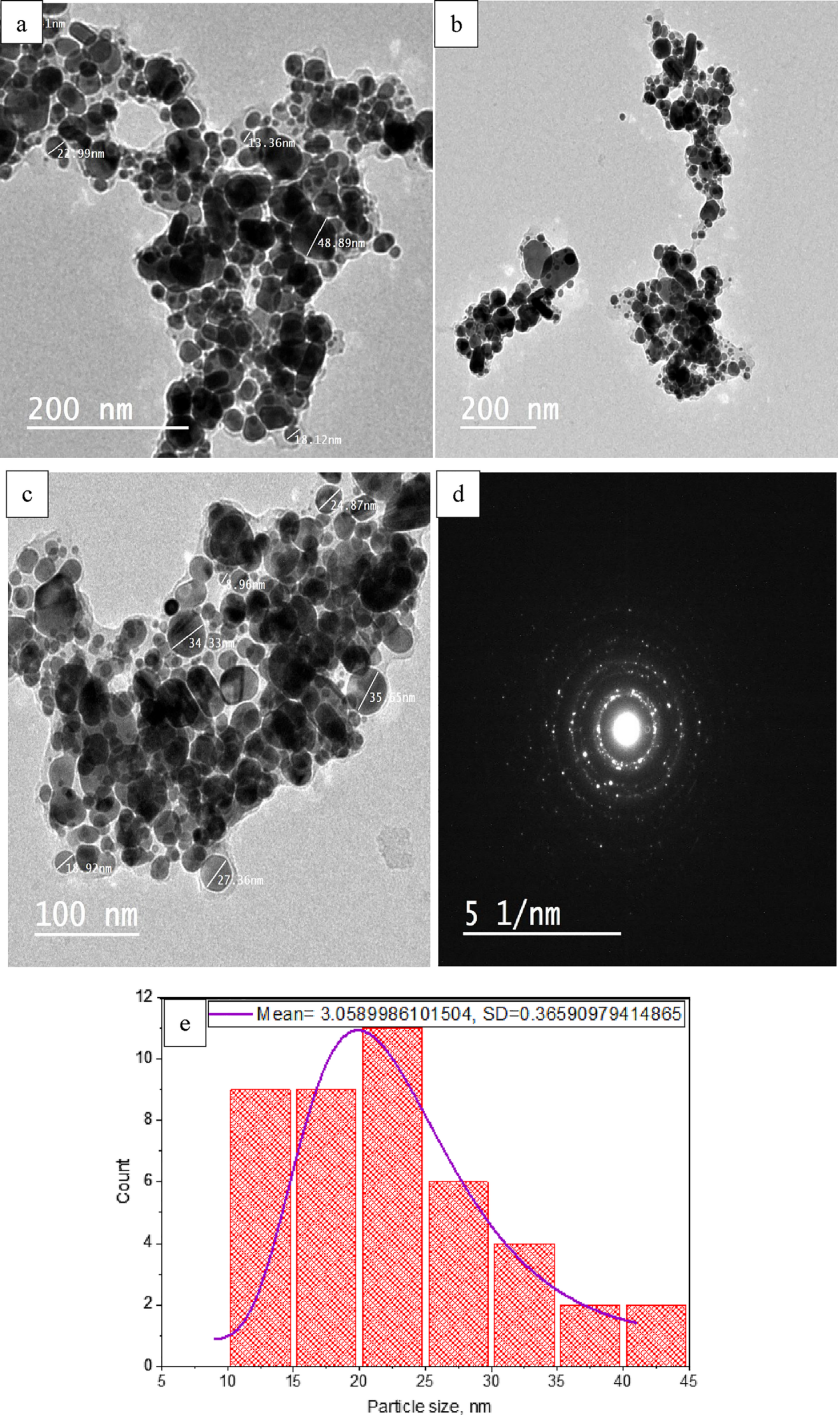
**a**–**c** TEM images of synthesized AgNPs, **d** selection electron diffraction pattern (SEAD) and **e** particle size distribution curve

TEM analysis was conducted to examine the morphology and size of the AgNPs under optimal conditions (pH 10, 70 °C, 2.5 mL of acacia leaf extract, and a stirring time of 2 h), as shown in Fig. 15a–d. The AgNPs were observed to have both rod-like and spherical shapes with smooth surfaces. The size distribution, displayed in Fig. 15e, indicates that the AgNPs range from 8 to 41 nm, predominantly clustering around 10–25 nm.

Table 2 gives a comparison of the silver nanoparticles synthesis condition produced using acacia raddiana leaf extract with the previous studies. It is clear that, the synthesized AgNPs in this study give results that are close to the earlier research as shown in Table 2. The particle size of the synthesized AgNPs in the present study has a small particle size range (8–41 nm) compared to the previous studies.

**Table 2:** Comparison between the current study and previous ones in the silver nanoparticle synthesis

Preparation method	Reducing agent	Conc. of AgNO_3_	Crystallite size, nm	pH	Temp., ^o^C	Particle size, nm	Λmax., nm	Ref
Green synthesis	Acacia raddiana leave extract (2.5 mL)	5 mM	35.5	pH10	70	8–41 (spherical)	423	Current study
Green synthesis	leaf extract of *Acer pentapomicum *(1 mL)	1 mM	9.5	pH6-7	35–55	19–25 (spherical)	450 nm	[113]
Green synthesis	*Acalypha hispida* leaf extract (0.5 mL)	1.75 mM	–	–	50	20–50 (spherical)	–	[114]
Microwave-assisted green synthesis	Pineapple leaves waste (6 mL)	20 mM	19	–	–	40–150 (hexagonal spherical shape)	400–450	[115]
Green synthesis	Aloefera (15%)	5 mM	–	–	60	34–102 (spherical)	420–490	[116]
Green synthesis	*Boswellia ovalifoliolata* (5 mL)	0.01 M	15	–		–	455	[117]
Green synthesis	*Clitoria ternatea* (5 mL)	0.1 M	20	pH9	–	–	420	[118]

### Sensor activity of AgNPs

#### Silver nanoparticle–metal ions interaction

**Fig. 16:**
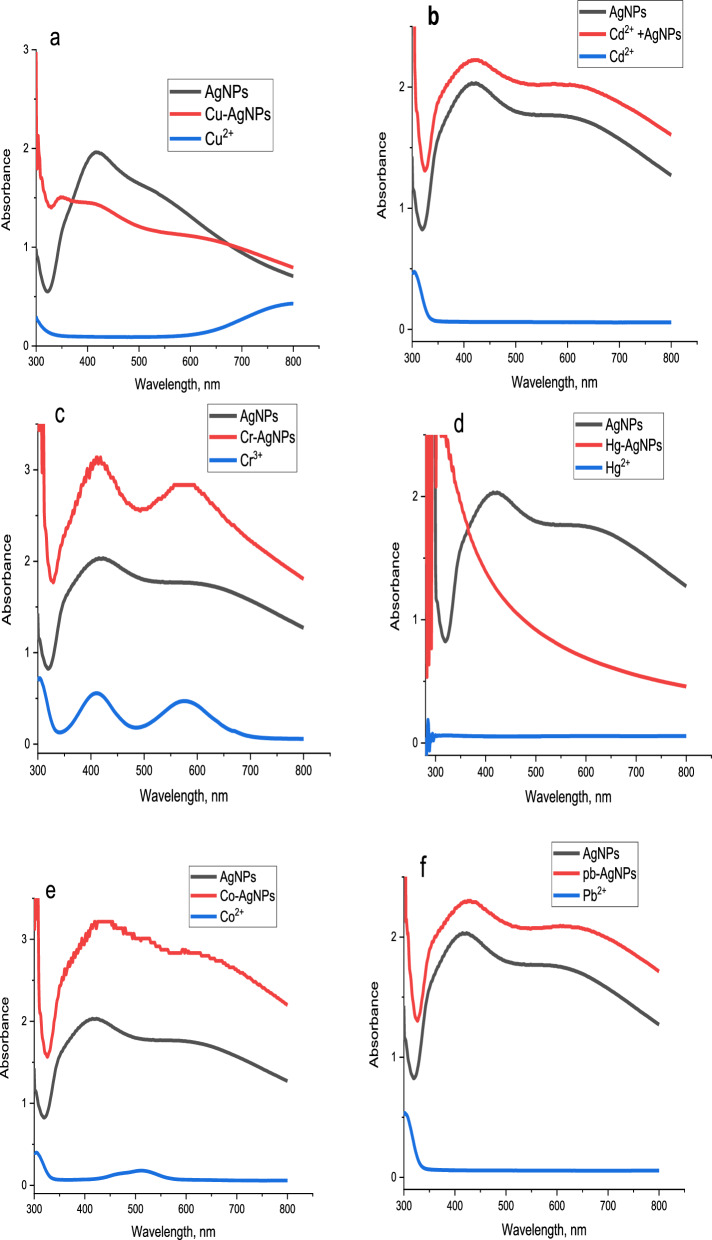
UV–Vis spectra of synthesized AgNPs Solution with **a** Cu^2+^, **b** Cd^2+^, **c** Cr^3+^, **d** Hg^2+^, **e** Co^2+^ and **f** Pb^2+^

**Fig. 17:**
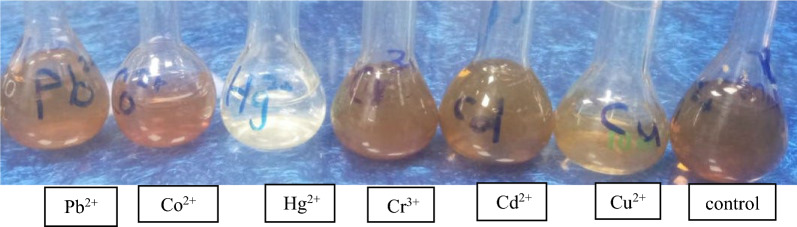
Colour of metals under study with AgNPs

It is widely known that prepared AgNPs have a brownish-yellow colour in an aqueous solution caused by the stimulating effect of surface plasmon resonance vibrations (SPR band) in AgNPs [119]. To evaluate the sensing abilities of the AgNPs to the metals Cu^2+^, Cd^2+^, Cr^3+^, Hg^2+^, Co^2+^ and Pb^2+^, 1 mL of 10^−3^ M metal ion was mixed with 1 mL of AgNPs suspension. As revealed in Figs. 16a–f and 17 as well as Table 3, upon addition of synthesized AgNPs to different solutions of metal ions, Cu^2+^ ions show a change in AgNPs colour from brownish-yellow to pale yellow resulting in a change in the absorbance band of AgNPs from 423 nm to 352 nm as shown in Fig. 16a. Figure 16b and c illustrate that Cd^2+^, as well as Cr^3+^ ions, do not noticeably affect the biologically synthesized AgNPs. The fast response of AgNPs to Hg^2+^ was observed when the solution colour changed from brownish-yellow to colourless, indicating the high selectivity and specificity of AgNPs for Hg^2+^ as illustrated in Fig. 16d. In addition, Fig. 16e and f, Co^2+^and Pb^2+^ ions show a change in AgNPs colour from brownish-yellow to pale red for Co^2+^ and yellowish red for Pb^2+^ resulting in a change in the absorbance band of AgNPs from 423 nm to 438 and 429 nm for Co^2+^and Pb^2+^, respectively. The interaction of AgNPs with these metal ions is explained as follows:

(i) The Cu^2+^ ion possesses a high standard reduction potential (Eo), indicating that copper ions can oxidize Ag^0^. This reaction causes a color shift in the AgNPs from yellowish-brown to a very pale yellow, which is likely due to Ag NP aggregation [120].
(ii) For the Hg^2+^ ion, the color disappearance in the silver nanoparticle solution is primarily attributed to a redox reaction involving Ag^0^ and Hg^2+^, which have standard potentials of 0.8 V (Ag^+^/Ag) and 0.85 V (Hg^2+^/Hg) respectively [121]. Two mechanisms are proposed for AgNPs' interaction with mercury. Initially, the addition of Hg^2+^ leads to the coating of AgNPs' external surface with Hg^0^, reducing absorbance and shifting the Surface Plasmon Resonance (SPR). Alternatively, the process could involve amalgam formation between AgNPs and Hg ions, a plausible reaction given the minor difference in electrochemical potentials between Hg^2+^ and AgNPs (0.8 V vs. 0.85 V), facilitating amalgam production and under-potential deposition [122, 123].
(iii) Regarding Co^2+^ ions, the introduction of Co (II) results in AgNP aggregation, changing their color from brownish-yellow to pale red. This change is attributed to covalent coordination bonds formed by catechol molecules (from the plant extract) on the AgNPs surface with Co^2+^ ions [124].
(iv) In the case of Pb^2+^ ions, the notable decrease and shift in the AgNPs' absorbance band post the addition of Pb (II) ion are likely due to AgNP aggregation. It is recognized that aggregation of nanoparticles leads to a shift in the Localized Surface Plasmon Resonance (LSPR) peak absorbance towards a higher wavelength maximum [125].

**Table 3:** Response of AgNPs as a sensor to heavy metals under investigation

Metals	Metal		Metal + AgNPs		AgNPs	
	λ, nm	Colour	λ, nm	Colour	λ, nm	Colour
Cu^2+^	797	Pale blue	352	Pale yellow		
Cd^2+^	228	Colourless	423	Brownish yellow	423	Brownish-yellow
Cr^3+^	411, 574	Dark violet	411, 574	Brownish yellow		
Hg^2+^	285	Colourless	No band	Colorless		
Co^2+^	304, 512	Red	438	Pale red		
Pb^2+^	285	Colourless	429	Yellowish red		

#### Effect of pH

**Fig. 18:**
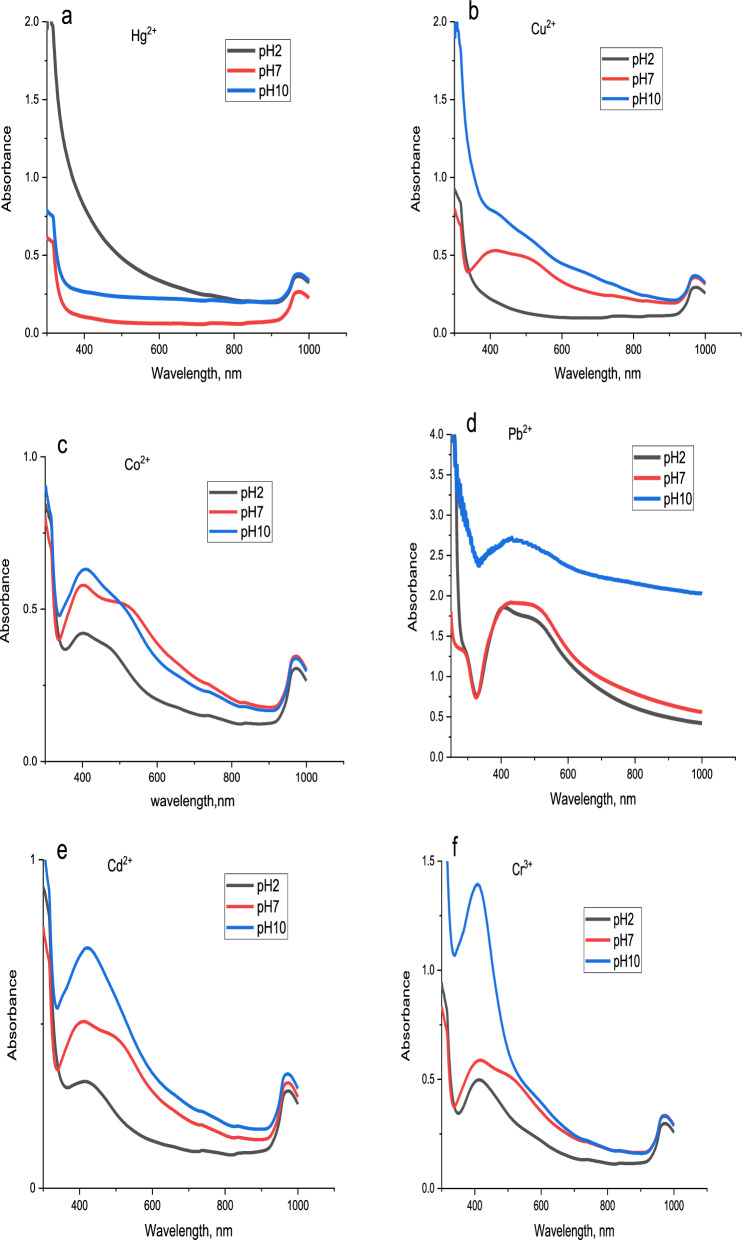
UV–Vis spectra green synthesized AgNPs solution with **a** Hg^2+^, **b** Cu^2+^, **c** Co^2+^, **d** Pb^2+^, **e** Cd^2+^ and **f** Cr^3+^ at different pH

For the six metals, the impact of pH was examined between the ranges of 2, 7, and 10 as shown in Fig. 18. Concerning mercury, the brownish-yellow colour of AgNPs disappeared at all studied pHs as illustrated in Fig. 18a. In the case of copper, Fig. 18b, a colour disappearance was observed at pH 2 and 10 as well a very pale yellow colour was obtained at pH 7. Figure 18c and d demonstrate that cobalt and lead show a shift in the absorbance band at pH 7 however at pH 2 and 10, give negligible results. Regarding Cd^2+^ and Cr^3+^ there are no significant results observed with pH change as shown in Fig. 18e and f).

#### Effect of AgNPs dose on Hg^2+^, Cu^2+^, Co^2+^ and Pb^2+^ detection

**Fig. 19:**
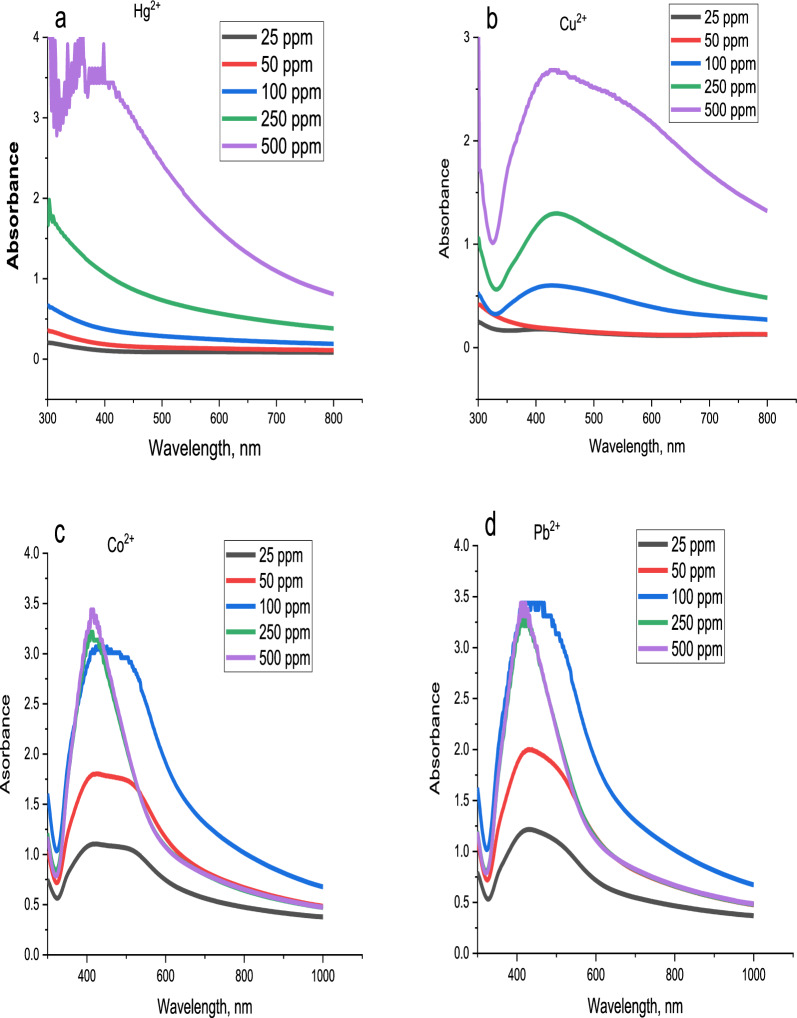
Effect of dose of AgNPs on **a** Hg^2+^, **b** Cu^2+^, **c** Co^2+^, and **d** Pb^2+^ detection

The effect of the AgNPs dose was studied for Hg^2+^, Cu^2+^, Co^2+^ and Pb^2+^ ions in the range 25, 50, 100, 250 and 500 ppm as shown in Fig. 19. The results indicated that mercury at doses 25, 50, 100 and 250 ppm disappear the absorbance band of AgNPs but at 500 ppm no change occurs as illustrated in Fig. 19a. According to copper, Fig. 19b, the peak of the AgNPs at 423 nm disappeared at 50 ppm and shifted to a lower wavelength (from 423 to 352 nm) at 25 ppm, but the other doses give the same curve with different absorbance values. For cobalt, Fig. 19c, doses 25, 50 and 100 ppm give a shift in the band (from 423 to 438 nm) with a difference in intensity of the absorbance band while, no change occurs in 250 and 500 ppm. In the case of lead, Fig. 19d, 25 and 50 ppm give absorbance bands at 429 nm but the doses 100, 250 and 500 give two absorbance bands at 413 and 429 nm.

#### Sensitivity and UV–vis studies

**Fig. 20:**
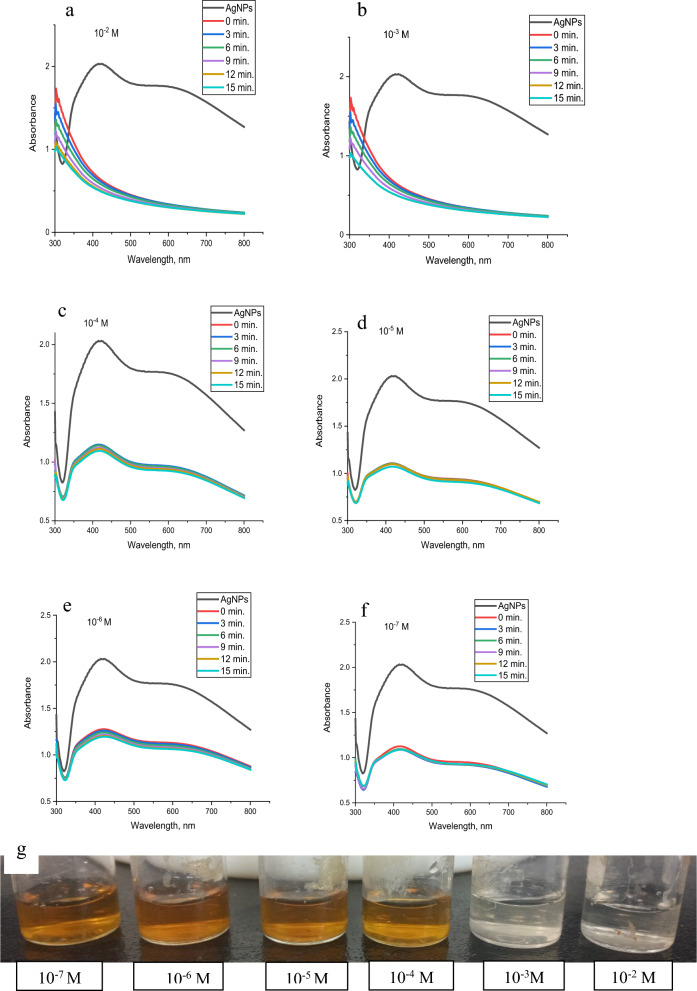
UV–Vis spectra of synthesized AgNPs in the presence of **a** 10^–2^, **b** 10^–3^, **c** 10^–4^, **d** 10^–5^, **e** 10^–6^ and **f** 10^–7^ M of Hg^2+^with time, Respectively and **g** the change of sensor colour with Hg^2+^ concentration

**Fig. 21:**
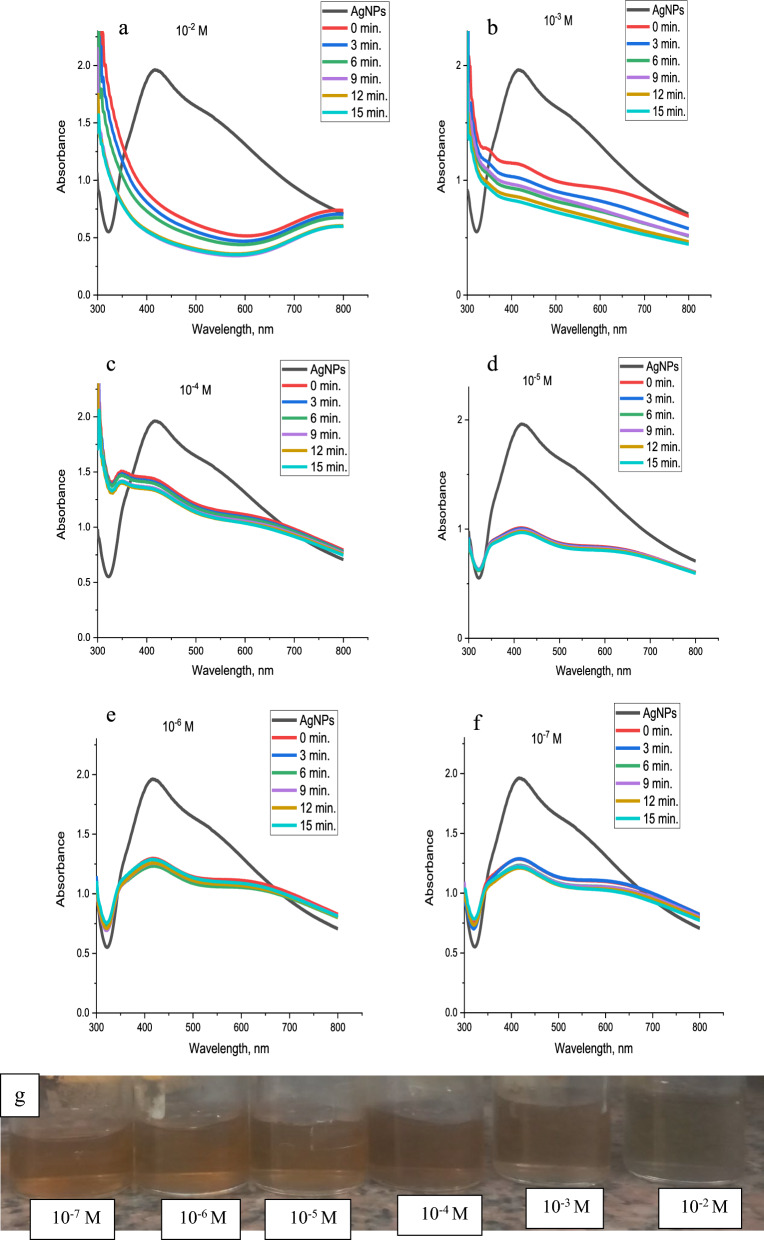
UV–Vis spectra of synthesized AgNPs in the presence of **a** 10^–2^, **b** 10^–3^, **c** 10^–4^, **d** 10^–5^, **e** 10^–6^ and **f** 10^–7^ M of Cu^2+^ with time, respectively and **g** the change of sensor colour with Cu^2+^ concentration

**Fig. 22:**
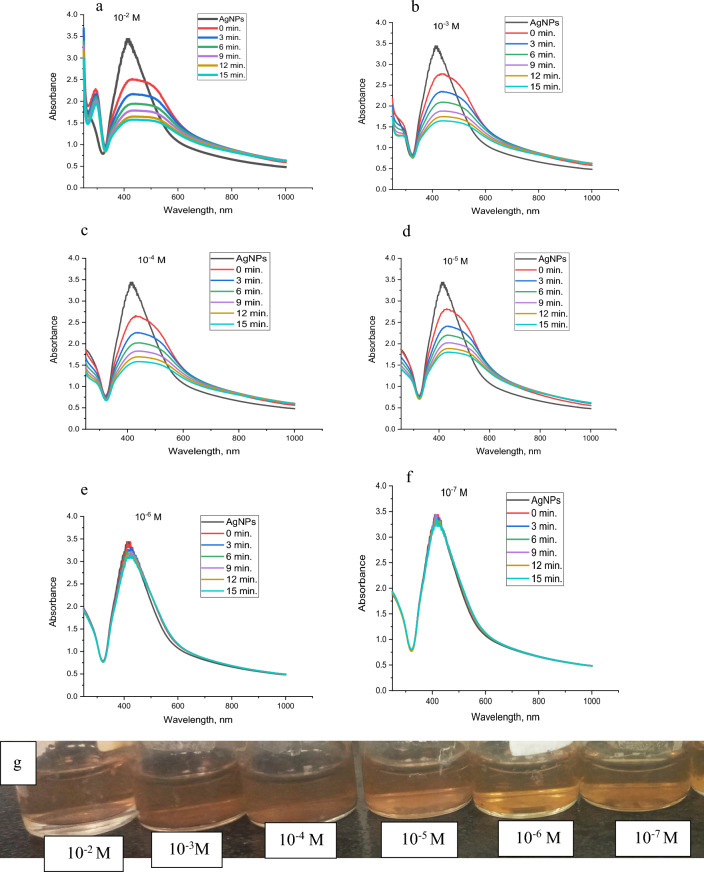
UV–Vis spectra of synthesized AgNPs in the presence of **a** 10^–2^, **b** 10^–3^, **c** 10^–4^, **d** 10^–5^, **e** 10^–6^ and **f** 10^–7^ M of Co^2+^with time, respectively and **g** the change of sensor colour with Co^2+^ concentration

**Fig. 23:**
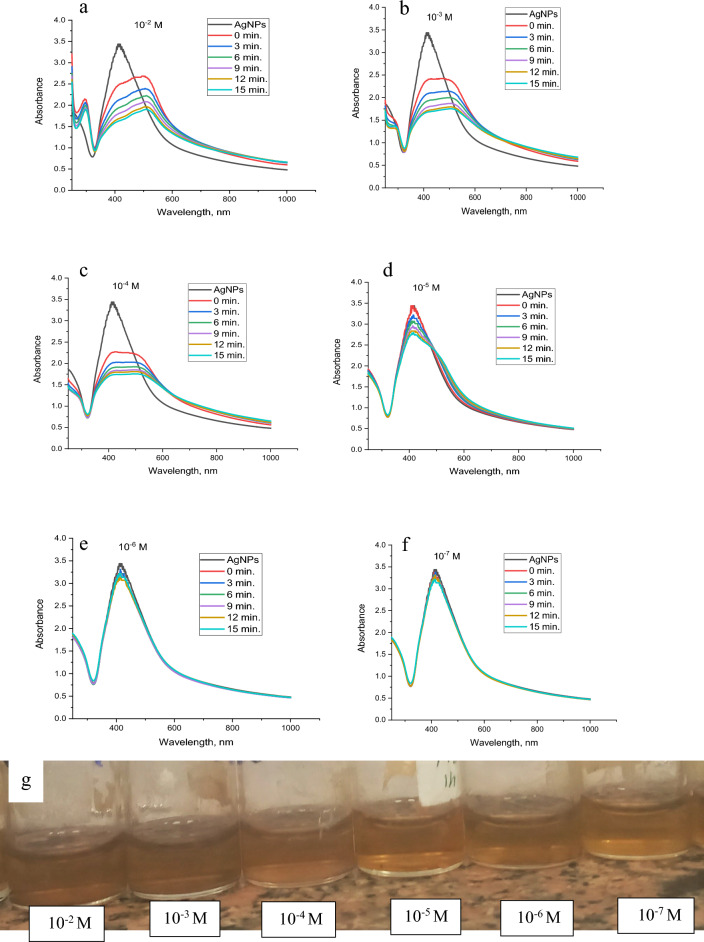
UV–Vis spectra of synthesized AgNPs in the presence of **a** 10^–2^, **b** 10^–3^, **c** 10^–4^, **d** 10^–5^, **e** 10^–6^ and **f** 10^–7^ M of Pb^2+^with time, respectively and **g** the change of sensor colour with Pb^2+^ concentration

By checking the colour transformation of the system and measuring the UV-vis absorbance reading, 1 mL of various concentrations of an aqueous solution of metal ions (10^-2^ to 10^-7^ M) was mixed with 1 mL of 4.6×10^-4^ M AgNPs solution at room temperature to evaluate the method's sensitivity and determine the lowest detectable concentration of Hg^2+^, Cu^2+^, Co^2+^, and Pb^2+^ in an aqueous solution. The general trend for the four studied metal ions (Hg^2+^, Cu^2+^, Co^2+^, and Pb^2+^) is identical as illustrated from Figs 20, 21, 22, 23 where raising the concentration of metal ions lowers the absorbance peaks of AgNPs.

From Fig. 20a and b, 1 × 10^−2^ and 1 × 10^−3^ M of mercury (II) transform the brownish yellow colour of AgNPs solution to colourless, whereas 1 × 10^−4^ M and 1 × 10^−5^ M, change the colour to pale yellow as indicated in Fig. 20c and d). On the other hand, several repeated experiments confirmed that 1 × 10^−6^ and 1 × 10^−7^ M of mercury (II) give little change of colour response to AgNPs as shown in Fig. 20e and f. The colour change of various concentrations of Hg^2+^ ions with AgNPs are illustrated in Fig. 20g.

For copper, Fig. 21a, at 1 × 10^-2^ the brownish-yellow colour of AgNPs solution changed to colourless and the absorbance band of AgNPs disappeared. 1 × 10^−3^ and 1 × 10^−4^ M changed the brownish yellow colour of AgNPs solution to pale yellow and a shift to a lower wavelength occurred from 423 to 352 nm as shown in Fig. 21b and c, whereas 1 × 10^−5^, 1 × 10^−6^ and 1 × 10^−7^ M, Fig. 20d–f, no colour alteration of AgNPs was observed. Figure 21g illustrates the colour of AgNPs with different concentrations of copper ions.

On behalf of cobalt, as illustrated in Fig. 22a–d, 1 × 10^-2^ M, 1 × 10^−3^ M, 1 × 10^−4,^ and 1 × 10^−5^ altered the colour of AgNPs from brownish yellow to pale red, whereas 1 × 10^−6^ and 1 × 10^−7^ M exhibit no variation in the AgNPs colour as shown in Fig. 22e and f. Figure 22g shows the colour change of AgNPs with different concentrations of cobalt.

In the case of lead, Fig. 23a–c, 1 × 10^−2^, 1 × 10^−3^ and 1 × 10^−4^ M change the brownish yellow colour of AgNPs solution to very pale red, while the 1 × 10^−5^ M converts the colour to yellow as illustrated in Fig. 23d. 1 × 10^−6^ and 1 × 10^−7^ M, Fig. 23e and f, maintain the colour of AgNPs. The colour change of AgNPs with lead is shown in Fig 23g.

The influence of time on the colour of the metal-AgNPs was studied in the range of 0-15 min. The four studied metals show fast response to AgNPs where the colour change is recorded at just metal addition (0 min.) to AgNPs. After 3, 6, 9, 12, and 15 min, the colour becomes constant for Hg^2+^ and Cu^2+^ whereas the colour of Pb^2+^ and Co^2+^ changed from pale red to pale yellow.

#### Dynamic range

**Fig. 24:**
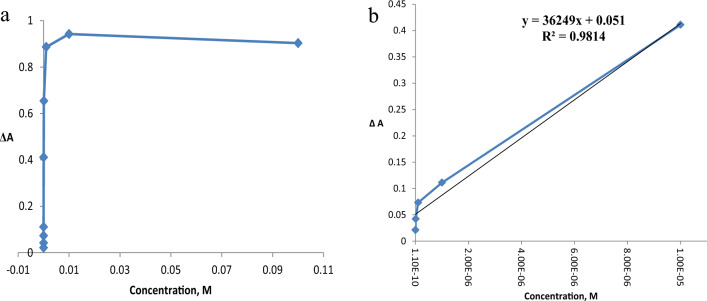
**a**, **b** Linear range of Hg^2+^ ion

**Fig. 25:**
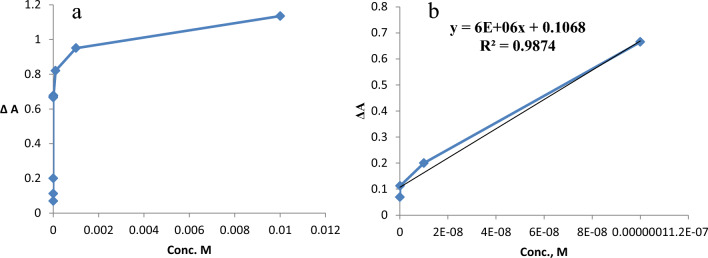
**a**, **b** Linear range of Cu^2+^ ion

**Fig. 26:**
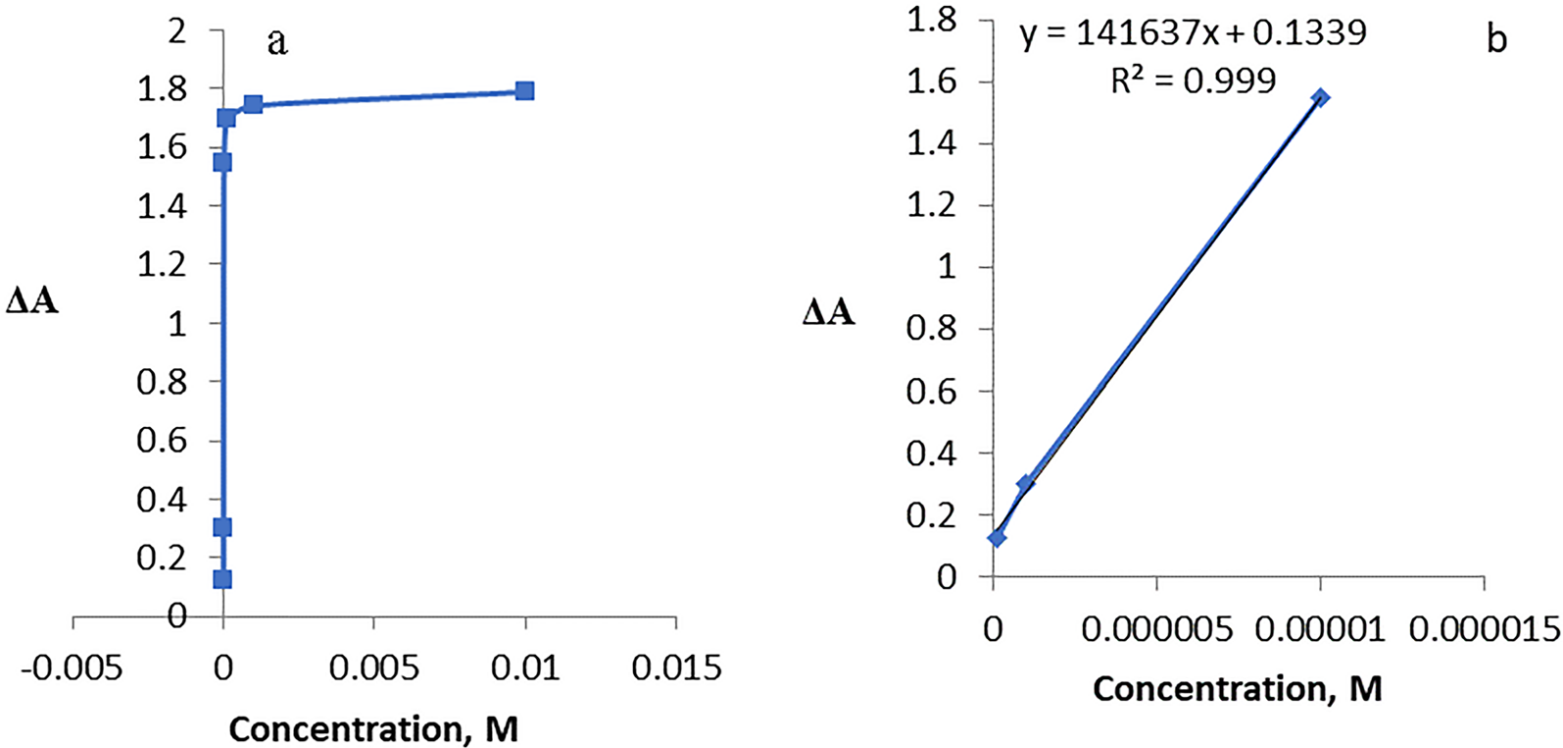
**a**, **b** Linear range of Pb^2+^ ion

**Fig. 27:**
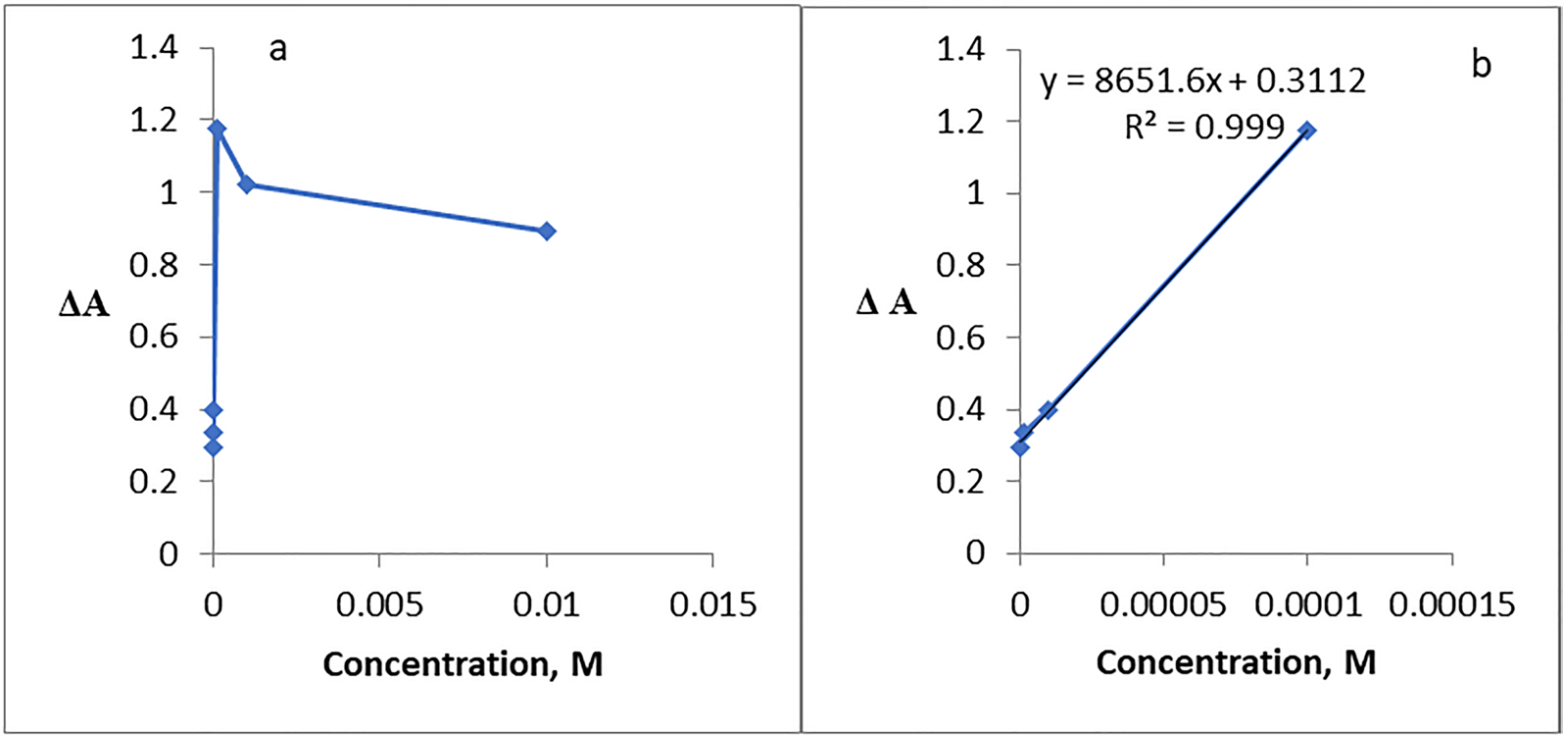
**a**, **b** Linear range of Co^2+^ ion

For a sensing device to be quantitatively useful, it must be demonstrated that its response changes depending on the analyst's concentration. A linear correlation can be seen in the nanosensor calibration plots for Hg^2+^, Cu^2+^, Pb^2+^ and Co^2+^ ions at low concentration ranges as shown in Figs. 24, 25, 26, 27.

Equation (6) was used to calculate the detection limit (LD) and quantification limit (LQ).

6 $$\mathrm{LD \,or\, LQ}=\frac{kSb}{m}$$

where S_b_ and m are the linear calibration curve's slope and standard deviations, respectively. For the determinations LD and LQ, the constant K is equal to 3 and 10, respectively [126]**.** The calculated LD and LQ for Hg^2+^, Cu^2+^, Pb^2+^ and Co^2+^ ions are described in Table 4.

**Table 4:** Calculated values of LD, LQ and Sandell's sensitivity

	Hg^2+^	Cu^2+^	Pb^2+^	Co^2+^
LD, M	1.322 × 10^–5^	1.37 × 10^–7^	1.63 × 10^–5^	1.34 × 10^–4^
LQ, M	4.4 × 10^–5^	4.5 × 10^–7^	5.44 × 10^–5^	4.486 × 10^–4^
Sandell’s sensitivity, µg cm^−2^	5.5 × 10^–3^	1.05 × 10^–5^	1.46 × 10^–3^	6.8 × 10^–3^

Sandell's pre-calculated sensitivity, Table 4, was described as the analyst concentration (in µg mL^−1^), which will absorb 0.001 in a path length cell 1 cm [126, 127] and which is expressed as g cm^−2^, calculated using Eq. (7):

7 $$S=n\frac{M}{\varepsilon }=\frac{M .\,Wt\times\, No \,of \,atoms}{Molar \,absorpitivity \,of \,coloured \,species}$$

The above equation can be interrupted as the following Eqs. (8, 9 and 10):

8 $${\text{S}}=\frac{{10}^{-3}}{{\upvarepsilon }_{s}}$$

9 $${\varepsilon }_{s}=\frac{\varepsilon }{M.\,Wt\, of\, determinant}\times 1000$$

10 $$\varepsilon =\frac{A}{C.d}$$

where ε_s_ is the specific absorptivity and its value in µg/cm corresponds to the determinant in a cuvette with an optical length of 1 cm, ε is the molar absorptivity, C is the molar concentration of the determinant, and d is path length (1 cm) [128]**.**

The absorbance signals of the AgNPs sensor with different concentrations of Hg^2+^ were investigated across a 10^–2^-10^–7^ M range. It was observed that Hg^2+^ showed linearity from 1 × 10^–7^–1 × 10^–4^ M (Fig. 24) with R^2^ 0.9814, LD 1.322 × 10^–5^, LQ 4.4 × 10^–5^ and Sandell’s sensitivity 5.5 × 10^–3^ µg cm^−2^.

For Cu^2+^, Fig. 25, linearity was observed from 1 × 10^–7^–1 × 10^–4^ M, with R^2^ 0.9874, LD 1.37 × 10^–7^ and LQ 4.5 × 10^–7^ and Sandell’s sensitivity 1.05 × 10^–5^ µg cm^−2^.

Lead ion, Fig. 26, exhibited a linearity from 1 × 10^–7^–1 × 10^–5^ M with R^2^ 0.999, LD 1.63 × 10^–5^, LQ 5.44 × 10^–5^ and Sandell’s sensitivity 1.46 × 10^–3^ µg cm^−2^.

In the case of cobalt, Fig. 27, exhibits linearity in the range 1 × 10^–7^– 1 × 10^–4^ M, where the R^2^ is 0.999, LD, 1.34 × 10^–4^, LQ 4.486 × 10^−4^ M and Sandell’s sensitivity 6.8 × 10^–3^ µg cm^−2^. For Hg^2+^ and Pb^2+^, 1 × 10^–5^ M is the concentration that saturate the AgNPs sensor, whereas for Cu^2+^ and Co^2+^ the concentration saturating the AgNPs sensor is 1 × 10^–7^ and 1 × 10^–4^ M, respectively.

#### Matrix effect

To examine the impact of the metals on each other, mixtures of metal–metal solutions were prepared by mixing 1 × 10^−3^ M of a given metal ion with 1 × 10^–3^ M of Cu^2+^, Cd^2+^, Co^2+^, Cr^3+^, Hg^2+^, Ni^2+^ or Pb^2+^. The total volume in all cases was adjusted to a graduated volume.

**Fig. 28:**
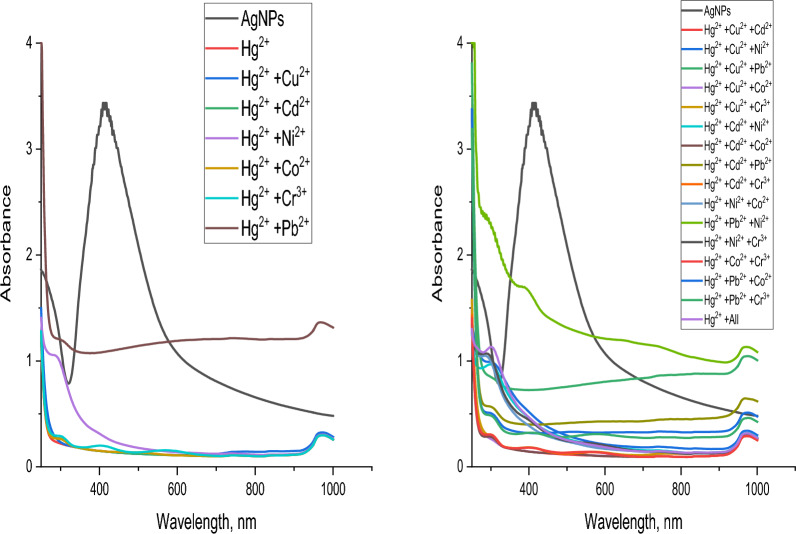
Sensitivity of AgNPs toward mercury with other metals under study

For Hg^2+^, Fig. 28, it can be detected that no interference occurs with all metals under study showing that the test technique has a very high level of sensitivity and selectivity for Hg^2+^

**Fig. 29:**
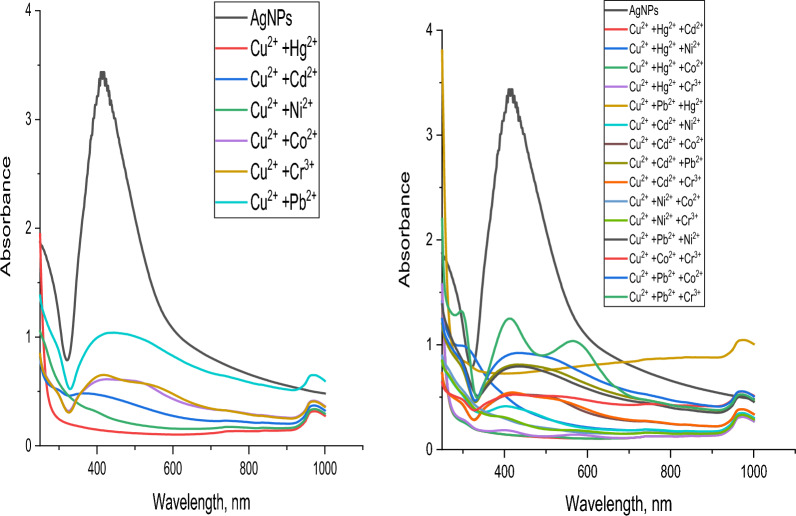
Sensitivity of AgNPs toward copper with other metals under study

Figure 29 shows the resulting data whenever Cu^2+^ is the given ion. It can be detected that only in the case of Cu^2+^/Ni^2+^ and Cu^2+^/Cd^2+^/Ni^2+^ mixtures no interference occurred.

**Fig. 30:**
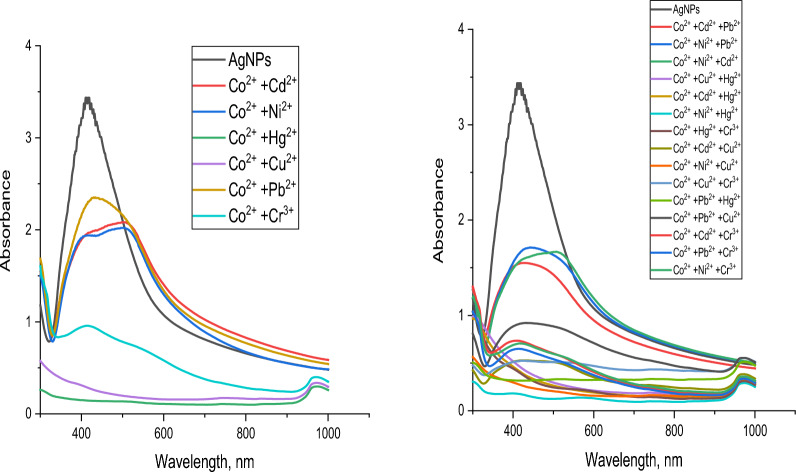
Sensitivity of AgNPs toward cobalt with other metals under study

Concerning Pb^2+^, Fig. 30, it can be detected that only in the case of Pb^2+^/Co^2+^ mixtures no interference occurred.

**Fig. 31:**
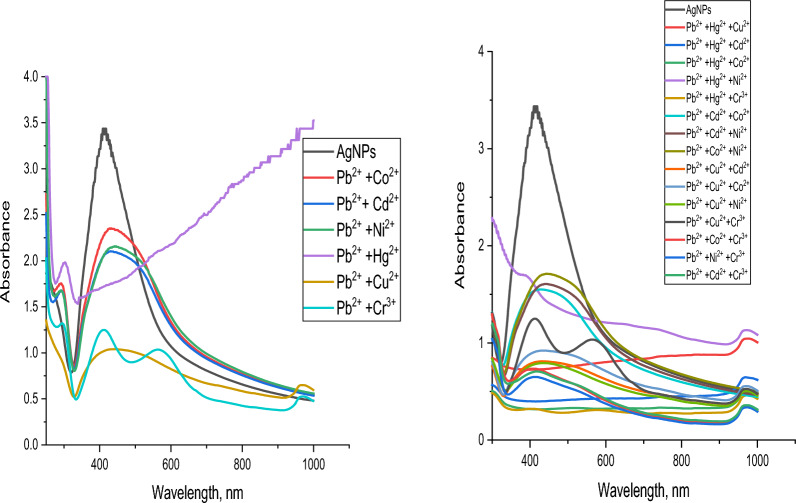
Sensitivity of AgNPs toward lead with other metals under study

Figure 31 represents the resulting data of Co^2+^ ions. It can be observed that all metals under study cause interference.

#### Comparison with previous literature

Table 5 presents a comparison of the current study with existing literature regarding the efficiency and capability of the synthesized AgNPs in detecting Hg^2+^, Pb^2+^, Co^2+^, and Cu^2+^ ions. The results show that the AgNPs synthesized in this study, without any modifications, offer a quicker response time for these four metal ions than those reported in other studies. Additionally, they exhibit relatively low detection limits, demonstrating the sensitivity of the prepared AgNPs towards Hg^2+^, Cu^2+^, Co^2+^, and Pb^2+^ ions. However, interference was observed in the detection of copper, lead, and cobalt ions in the presence of other metals, which represents a limitation of this study.

**Table 5:** Analytical performance of the AgNPs sensor with previously reported sensors for the Hg^2+^, Pb^2+^, Co^2+^ and Cu^2+^

Element	LD	LQ	R^2^	Conc. range	Colorimetric sensor	Response time	Matrix effect	Refs.
Hg^2+^	1.322 × 10^−5^ M	4.4 × 10^−5^ M	0.9814	10^–2^–10^−7^ M	AgNPs without modification	Just added	No interference	Current study
	8 × 10^–7^ M	–	0.9956	1-70 µM	AgNPs without modification	5 min	No interferences	[126]
	28 ppm	85 ppm	0.965	7–63 ppm	AgNPs without modification	1 min	No interferences	[129]
	15 ppb	–	0.995	10–1000 ppb	AgNPs without modification	5 min	–	[130]
	0.5 mg/L	1.69 mg/L	0.989	10–80 mg/L	AgNPs without modification	30 s	–	[131]
	1.8 nmol L-1	–	–	0.005 to 41 μmol L^−1^	Boronic acid functionalized MoS2 quantum dot	–	–	[132]
Pb^2+^	1.63 × 10^–5^	5.44 × 10^–5^	0.999	10^–2^–10^–7^ M	AgNPs without modification	Just added	only in the case of Pb^2+/^Co^2+^ mixtures, no interference occurred	Current study
	2.0 × 10^−7^ M	–	0.9959	1–90 μM	AgNPs Without modification	5 min	No interferences	[126]
	0.03 × 10^−2^ µg/L	–	0.97	0.10–10 µg/L	Modify (silver (Ag)–gold (Au) alloy nanoparticle (NP)–aptamer-modified glassy carbon electrode (GCE))	–	No interferences except with Cd^2+^	[133]
	0.056 µmol L^−1^	–	–	0.19–1.29 µmol L^−1^	Modify (citrate-capped Ag nanoparticles)	–	–	[134]
	0.1 μM	–	0.996	0.5—25 μM	AgNPs without modification	–	–	[135]
	0.05 nM	–	–	0.01-100 µM	GR-5 DNAzyme based Pb ion strip biosensor	–	–	[136]
Co^2+^	1.34 × 10^–4^	4.486 × 10^–4^	0.999	10^–2^-10^−7^ M	AgNPs without modification	Just added	Cause interferences	Current study
	0.1 μM	0.3 μM	0.9984	0.1–5 μM	Povidone capped silver nanoparticles	4–5 min	–	[137]
	7.0 μM	–	0.99431	05–100 μM	Modify triazole–carboxyl agnps	5 min	–	[138]
	0.16	0.55 mM	–	1–30 mM	Lignin-functionalized silver nanoparticles	–	–	[139]
	0.68 µM	–	0.9957	1.7–20 µM	AgGSH silver nanoparticle-glutathione with cysteine modify	–	–	[140]
Cu^2+^	1.37 × 10^–7^ M	4.5 × 10^–7^ M	0.9874	10^–2^–10^–7^ M	AgNPs without modification	Just added	only in the case of Cu^2+^/Ni^2+^ and Cu^2+^/Cd^2+^/Ni^2+^ mixtures no interference occurred	Current study
	1.7 μM	–	0.9795	2.5 µM–1 mM	Carrageenan-silver nanoparticles	3 min	–	[141]
	0.16 µM	–	0.973	0.08–1.44 µM	Casein peptide-functionalized silver nanoparticles	–	–	[142]
	2.5 × 10^−8^ M	–	0.998	10^−7^–10^−4^ M	4-mercaptobenzoic acid modified silver nanoparticles	–	–	[143]
	2 nM	–	0.9965	20 to 0.002 μM	AgNPs stabilized with sodium pyrophosphate (Na4P_2_O_7_) and hydroxypropylmethylcellulose (HPMC)	10 min	No interferences	[144]
	1 μM	–	–	1–100 μM	Specifically modified *Saccharomyces cerevisiae* strain immobilized in alginate beads	–	–	[145]

### Real sample analysis

Table 6 shows the efficiency of the AgNPs as colouring sensor for the heavy metals under study in wastewater samples. The percentage efficiency of AgNPs toward Pb is 42.43%, Cu is 100.72% and Co is 42.33%. While mercury isn’t found in the sample.

**Table 6:** The efficiency of the AgNPs as colouring sensor for the heavy metals under study in wastewater samples (KIMA)

Element	Actual (ppm)	Found using sensor (ppm)	AgNPs efficiency as colouring sensor
Hg^2+^	Not found	–	–
Cu^2+^	0.0063	0.0063	100.72%
Pb^2+^	0.0004	0.0001697	42.43%
Co^2+^	0.0064	0.0027	42.33%

## Conclusion

The *acacia raddiana* leaf extract is rich in secondary chemical constituents (phenols, tannins, alkaloids, saponins and flavonoids). These chemicals behave as reducing and stabilizing reagents for synthesized AgNPs. The influence of leave extract volume, pH, temperature and time on the interaction response and morphology of the synthesized AgNPs are examined. The AgNPs synthesized at pH10, for 2 h with extract volume 2.5 mL and temperature 70 °C give sharp peaks and are more crystalline than the other samples. The AgNPs surface acquired a negative charge according to the zeta potential results which was found to be − 32 mM. The AgNPs have mostly a spherical structure with particle sizes between 8 and 41 nm. The synthesized AgNPs can be utilized to detect mercury, copper, lead and cobalt with LD and LQ 1.322 × 10^–5^ M and 4.4 × 10^–5^ M for mercury, 1.37 × 10^–7^ M and 4.5 × 10^–7^ M for copper, 1.63 × 10^–5^ M, 5.44 × 10^–5^ M for lead and 1.34 × 10^–4^ M, 4.486 × 10^–4^ M for cobalt, respectively. Hg^2+^ can be detected in the presence of all metals under study individually or together. Co^2+^ cannot be detected in the presence of all metals under study individually or together. Cu^2+^ can be detected in the presence of Ni^2+^ individual and Cd^2+^ + Ni^2+^ together. Pb^2+^ can be detected in the presence of Co^2+^ only. Future studies should explore additional modifications to AgNPs to enhance the sensitivity of colorimetric detection of heavy metals and to reduce potential interferences.

## Data Availability

All data included in this study are present in this published article.

## References

[1] Seifipour R, Nozari M, Pishkar L (2020). Green synthesis of silver nanoparticles using *Tragopogon collinus* leaf extract and study of their antibacterial effects. J Inorg Organomet Polym Mater.

[2] Salem SS, Fouda A (2021). Green synthesis of metallic nanoparticles and their prospective biotechnological applications: an overview. Biol Trace Elem Res.

[3] Malik S, Muhammad K, Waheed Y (2023). Nanotechnology: a revolution in modern industry. Molecules.

[4] Piracha S, Saleem S, Momil G, Anjum A, Yaseen Z (2021). Nanoparticle: role in chemical industries, potential sources and chemical catalysis applications. Sch Int J Chem Mater Sci.

[5] Anselmo AC, Mitragotri S (2019). Nanoparticles in the clinic: an update. Bioeng Transl Med..

[6] Kumar R, Kumar M, Luthra G (2023). Fundamental approaches and applications of nanotechnology: a mini review. Mater Today Proc.

[7] Cuong HN, Pansambal S, Ghotekar S, Oza R, Hai NTT, Viet NM (2022). New frontiers in the plant extract mediated biosynthesis of copper oxide (CuO) nanoparticles and their potential applications: a review. Environ Res.

[8] Kumar R, Raizada P, Khan AAP, Nguyen V-H, Van Le Q, Ghotekar S (2022). Recent progress in emerging BiPO_4_-based photocatalysts: synthesis, properties, modification strategies, and photocatalytic applications. J mater sci technol.

[9] Pandit C, Roy A, Ghotekar S, Khusro A, Islam MN, Emran TB (2022). Biological agents for synthesis of nanoparticles and their applications. J King Saud Univ Sci..

[10] Ghotekar S, Pansambal S, Bilal M, Pingale SS, Oza R (2021). Environmentally friendly synthesis of Cr_2_O_3_ nanoparticles: characterization, applications and future perspective─a review. Case Stud Chem Environ Eng..

[11] Narwal N, Katyal D, Kataria N, Rose PK, Warkar SG, Pugazhendhi A (2023). Emerging micropollutants in aquatic ecosystems and nanotechnology-based removal alternatives: a review. Chemosphere.

[12] Pansambal S, Oza R, Borgave S, Chauhan A, Bardapurkar P, Vyas S (2023). Bioengineered cerium oxide (CeO_2_) nanoparticles and their diverse applications: a review. Appl Nanosci.

[13] Ghotekar S, Pansambal S, Lin K-YA, Pore D, Oza R (2023). Recent advances in synthesis of CeVO_4_ nanoparticles and their potential scaffold for photocatalytic applications. Top Catal.

[14] Faisal S, Khan MA, Jan H, Shah SA, Shah S, Rizwan M (2020). Edible mushroom (*Flammulina velutipes*) as biosource for silver nanoparticles: from synthesis to diverse biomedical and environmental applications. Nanotechnology.

[15] Mohammad ZH, Ahmad F, Ibrahim SA, Zaidi S (2022). Application of nanotechnology in different aspects of the food industry. Discov Food..

[16] Singh A, Gaud B, Jaybhaye S (2020). Optimization of synthesis parameters of silver nanoparticles and its antimicrobial activity. Mater Sci Energy Technol.

[17] Usman AI, Aziz AA, Noqta OA (2019). Green sonochemical synthesis of gold nanoparticles using palm oil leaves extracts. Mater Today: Proc.

[18] Khan AA, Fox EK, Górzny MŁ, Nikulina E, Brougham DF, Wege C (2013). pH control of the electrostatic binding of gold and iron oxide nanoparticles to tobacco mosaic virus. Langmuir.

[19] Husain S, Nandi A, Simnani FZ, Saha U, Ghosh A, Sinha A (2023). Emerging trends in advanced translational applications of silver nanoparticles: a progressing dawn of nanotechnology. J Funct Biomater..

[20] Singh A, Jain D, Upadhyay MK, Khandelwal N, Verma HN (2010). Green synthesis of silver nanoparticles using *Argemone mexicana* leaf extract and evaluation of their antimicrobial activities. Dig J Nanomater Bios.

[21] Sudarman F, Shiddiq M, Armynah B, Tahir D. Silver nanoparticles (AgNPs) synthesis methods as heavy-metal sensors: a review. Int J Environ Sci Technol. 2023:1–18.

[22] Ajitha B, Reddy YAK, Reddy PS (2014). Biogenic nano-scale silver particles by *Tephrosia purpurea* leaf extract and their inborn antimicrobial activity. Spectrochim Acta A: Mol Biomol Spectrosc.

[23] Güzel R, Erdal G (2018). Synthesis of silver nanoparticles.

[24] Yaqoob AA, Umar K, Ibrahim MNM (2020). Silver nanoparticles: various methods of synthesis, size affecting factors and their potential applications–a review. Appl Nanosci.

[25] Mughal SS. Role of silver nanoparticles in colorimetric detection of biomolecules. Authorea Preprints. 2022.

[26] Khan MI, Shah S, Faisal S, Gul S, Khan S, Abdullah (2022). Monotheca buxifolia driven synthesis of zinc oxide nano material its characterization and biomedical applications. Micromachines..

[27] Xu L, Wang Y-Y, Huang J, Chen C-Y, Wang Z-X, Xie H (2020). Silver nanoparticles: synthesis, medical applications and biosafety. Theranostics..

[28] Nguyen NPU, Dang NT, Doan L, Nguyen TTH (2023). Synthesis of silver nanoparticles: from conventional to ‘modern’ methods—a review. Processes.

[29] Zafar S, Faisal S, Jan H, Ullah R, Rizwan M, Abdullah (2022). Development of iron nanoparticles (FeNPs) using biomass of enterobacter: its characterization, antimicrobial, anti-Alzheimer’s, and enzyme inhibition potential. Micromachines..

[30] Suriati G, Mariatti M, Azizan A (2014). Synthesis of silver nanoparticles by chemical reduction method: effect of reducing agent and surfactant concentration. Int J Autom Mech Eng.

[31] Muthuvel A, Jothibas M, Manoharan C (2020). Synthesis of copper oxide nanoparticles by chemical and biogenic methods: photocatalytic degradation and in vitro antioxidant activity. Nanotechnol Environ Eng.

[32] Malik MA, Wani MY, Hashim MA (2012). Microemulsion method: a novel route to synthesize organic and inorganic nanomaterials: 1st nano update. Arab J Chem.

[33] dos Santos MA, Paterno LG, Moreira SGC, Sales MJA (2019). Original photochemical synthesis of Ag nanoparticles mediated by potato starch. SN Appl Sci.

[34] Naganthran A, Verasoundarapandian G, Khalid FE, Masarudin MJ, Zulkharnain A, Nawawi NM (2022). Synthesis, characterization and biomedical application of silver nanoparticles. Materials.

[35] Bankar A, Joshi B, Kumar AR, Zinjarde S (2010). Banana peel extract mediated novel route for the synthesis of silver nanoparticles. Colloids Surf A: Physicochem Eng Asp.

[36] AH Alrajhi, NM Ahmed. Green synthesis of zinc oxide nanoparticles using salvia officinalis extract. Handbook of green and sustainable nanotechnology: fundamentals, developments and applications. Springer; 2023, p. 1–21.

[37] Shabatina T, Bochenkov V. Smart nanosystems for biomedicine, optoelectronics and catalysis: BoD–Books on demand; 2020.

[38] Srikar SK, Giri DD, Pal DB, Mishra PK, Upadhyay SN (2016). Green synthesis of silver nanoparticles: a review. Green Sustain Chem..

[39] Hussain T, Faisal S, Rizwan M, Zaman N, Iqbal M, Iqbal A (2022). Green synthesis and characterization of copper and nickel hybrid nanomaterials: investigation of their biological and photocatalytic potential for the removal of organic crystal violet dye. J Saudi Chem Soc.

[40] Ahmed S, Ahmad M, Swami BL, Ikram S (2016). A review on plants extract mediated synthesis of silver nanoparticles for antimicrobial applications: a green expertise. J Adv Res.

[41] Shah S, Shah SA, Faisal S, Khan A, Ullah R, Ali N (2022). Engineering novel gold nanoparticles using Sageretia thea leaf extract and evaluation of their biological activities. J Nanostruct Chem..

[42] Ge L, Li Q, Wang M, Ouyang J, Li X, Xing MMQ (2014). Nanosilver particles in medical applications: synthesis, performance, and toxicity. Int J Nanomed.

[43] Deivanathan SK, Prakash JTJ (2023). Bio-synthesis of silver nanoparticles using leaf extract of *Rhaphidophora pertusa* and its characterization, antimicrobial, antioxidant and cytotoxicity activities. Res Chem Intermed.

[44] Jain N, Jain P, Rajput D, Patil UK (2021). Green synthesized plant-based silver nanoparticles: therapeutic prospective for anticancer and antiviral activity. Micro Nano Syst Lett..

[45] Bard A (2017). Standard potentials in aqueous solution.

[46] Bhati M (2023). Biogenic synthesis of metallic nanoparticles: principles and applications. Mater Today: Proc.

[47] Christensen L, Vivekanandhan S, Misra M, Kumar Mohanty A (2011). Biosynthesis of silver nanoparticles using *Murraya koenigii* (curry leaf): an investigation on the effect of broth concentration in reduction mechanism and particle size. Adv Mater Lett.

[48] Zarei Z, Razmjoue D, Karimi J (2020). Green synthesis of silver nanoparticles from *Caralluma tuberculata* extract and its antibacterial activity. J Inorg Organomet Polym Mater.

[49] Gahlawat G, Choudhury AR (2019). A review on the biosynthesis of metal and metal salt nanoparticles by microbes. RSC Adv.

[50] El Khoury E, Abiad M, Kassaify ZG, Patra D (2015). Green synthesis of curcumin conjugated nanosilver for the applications in nucleic acid sensing and anti-bacterial activity. Colloids Surf B.

[51] de Arrifano GP, Augusto-Oliveira M, Lopes-Araújo A, Santos-Sacramento L, Macchi BM, Nascimento JLM (2023). Global human threat: the potential synergism between mercury intoxication and COVID-19. Int J Environ Res Public Health.

[52] Sowmyya T, Lakshmi GV (2018). Soymida febrifuga aqueous root extract maneuvered silver nanoparticles as mercury nanosensor and potential microbicide. World Sci News..

[53] Forough M, Farhadi K (2010). Biological and green synthesis of silver nanoparticles. Turkish J Eng Environ Sci..

[54] Zhang Z, Zhao Z, Fang Q, Qiao R, Zhang T (2023). Extracellular polymeric substances enhance dissolution and microbial methylation of mercury sulfide minerals. Environ Sci Process Impacts.

[55] Zhang D, Ma X-I, Gu Y, Huang H, Zhang G-W (2020). Green synthesis of metallic nanoparticles and their potential applications to treat cancer. Front Chem.

[56] Bahrulolum H, Nooraei S, Javanshir N, Tarrahimofrad H, Mirbagheri VS, Easton AJ (2021). Green synthesis of metal nanoparticles using microorganisms and their application in the agrifood sector. J Nanobiotechnol.

[57] Jarzyńska G, Falandysz J (2011). The determination of mercury in mushrooms by CV-AAS and ICP-AES techniques. J Environ Sci Health A.

[58] Lin Y-W, Peng S-Y, Lee W-H, Lin Y-Y, Hung M-J, Lin K-L (2023). Characterization of Cu^2+^ adsorption for eco-hydroxyapatite derived from limestone sludge via hydrothermal synthesis. J Mater Cycles Waste Manag.

[59] Martín-Yerga D, González-García MB, Costa-García A (2013). Electrochemical determination of mercury: a review. Talanta.

[60] Cao X, Li W, Song S, Wang C, Khan K (2023). Source apportionment and risk assessment of soil heavy metals around a key drinking water source area in northern China: multivariate statistical analysis approach. Environ Geochem Health.

[61] Singh PK, Shikha D, Saw S (2023). Evaluation of potential toxic heavy metal contamination in soil, fly ash, vegetables and grain crops along with associated ecological and health risk assessment of nearby inhabitants of a thermal power station in Jharkhand (India). Environ Sci Pollut Res.

[62] Farhadi K, Forough M, Molaei R, Hajizadeh S, Rafipour A (2012). Highly selective Hg^2+^ colorimetric sensor using green synthesized and unmodified silver nanoparticles. Sens Actuators B Chem.

[63] Bača P, Vanýsek P (2023). Issues concerning manufacture and recycling of lead. Energies.

[64] Kulkarni SK, Kulkarni SK (2015). Nanotechnology: principles and practices.

[65] Zhang B-Y, Shi L, Ma X-Y, Liu L, Fu Y, Zhang X-F (2023). Advances in the functional nucleic acid biosensors for detection of lead ions. Crit Rev Anal Chem.

[66] Tran QH, Le A-T (2013). Silver nanoparticles: synthesis, properties, toxicology, applications and perspectives. Adv Nat Sci: Nanosci Nanotechnol..

[67] Bruna T, Maldonado-Bravo F, Jara P, Caro N (2021). Silver nanoparticles and their antibacterial applications. Int J Mol Sci.

[68] Sangaonkar GM, Desai MP, Dongale TD, Pawar KD (2020). Selective interaction between phytomediated anionic silver nanoparticles and mercury leading to amalgam formation enables highly sensitive, colorimetric and memristor-based detection of mercury. Sci Rep.

[69] Atasoy M, Yildiz D, Kula İ, Vaizoğullar Aİ (2023). Determination and speciation of methyl mercury and total mercury in fish tissue samples by gold-coated W-coil atom trap cold vapor atomic absorption spectrometry. Food Chem.

[70] Hossain N, Islam MA, Chowdhury MA (2022). Synthesis and characterization of plant extracted silver nanoparticles and advances in dental implant applications. Heliyon..

[71] Ozalp O, Soylak M (2023). Ag modified ZnO nanoflowers for the dispersive micro-solid-phase extraction of lead (II) from food and water samples prior to its detection with high-resolution continuum source flame atomic absorption spectrometry. Talanta.

[72] Wilschefski SC, Baxter MR (2019). Inductively coupled plasma mass spectrometry: introduction to analytical aspects. Clin Biochem Rev.

[73] Abdel-Raouf N, Al-Enazi NM, Ibraheem IBM, Alharbi RM, Alkhulaifi MM (2019). Biosynthesis of silver nanoparticles by using of the marine brown alga *Padina pavonia* and their characterization. Saudi J Biol Sci..

[74] Yuan L, Liang M, Hummel M, Shao C, Lu S (2023). Rational design copper nanocluster-based fluorescent sensors towards heavy metal ions: a review. Chemosensors..

[75] Balali-Mood M, Naseri K, Tahergorabi Z, Khazdair MR, Sadeghi M (2021). Toxic mechanisms of five heavy metals: mercury, lead, chromium, cadmium, and arsenic. Front Pharmacol.

[76] Ding Q, Li C, Wang H, Xu C, Kuang H (2021). Electrochemical detection of heavy metal ions in water. Chem Commun.

[77] Verkhovskii R, Kozlova A, Atkin V, Kamyshinsky R, Shulgina T, Nechaeva O (2019). Physical properties and cytotoxicity of silver nanoparticles under different polymeric stabilizers. Heliyon..

[78] Wasilewska A, Klekotka U, Zambrzycka M, Zambrowski G, Święcicka I, Kalska-Szostko B (2023). Physico-chemical properties and antimicrobial activity of silver nanoparticles fabricated by green synthesis. Food Chem.

[79] Alberti G, Zanoni C, Magnaghi LR, Biesuz R (2021). Gold and silver nanoparticle-based colorimetric sensors: new trends and applications. Chemosensors..

[80] Ismail M, Khan MI, Akhtar K, Khan MA, Asiri AM, Khan SB (2018). Biosynthesis of silver nanoparticles: a colorimetric optical sensor for detection of hexavalent chromium and ammonia in aqueous solution. Physica E.

[81] Prosposito P, Burratti L, Bellingeri A, Protano G, Faleri C, Corsi I (2019). Bifunctionalized silver nanoparticles as Hg2+ plasmonic sensor in water: synthesis, characterizations, and ecosafety. Nanomaterials.

[82] Suvarapu LN, Baek S-O (2017). Recent studies on the speciation and determination of mercury in different environmental matrices using various analytical techniques. Int J Anal Chem..

[83] Thiyagarajan S, Kanchana S. Green synthesis of silver nanoparticles using leaf extracts of *Mentha arvensis* Linn. and demonstration of their in vitro antibacterial activities. Brazilian J Pharm Sci. 2022;58.

[84] Akinsipo OB, Alayande SO, Mustapha TO, Abayomi ME, Adeloye D, Osinubi AD (2023). Green synthesis of silver nanoparticles using *Lagenaria breviflora* aqueous leaves extract. CaJoST..

[85] Pramila DM, Xavier R, Marimuthu K, Kathiresan S, Khoo ML, Senthilkumar M (2012). Phytochemical analysis and antimicrobial potential of methanolic leaf extract of peppermint (*Mentha piperita*: Lamiaceae). J Med Plants Res..

[86] Rice-Evans C (2004). Flavonoids and isoflavones: absorption, metabolism, and bioactivity. Free Radic Biol Med.

[87] Gurunathan S, Han JW, Kwon D-N, Kim J-H (2014). Enhanced antibacterial and anti-biofilm activities of silver nanoparticles against Gram-negative and Gram-positive bacteria. Nanoscale Res Lett.

[88] Ferreira SLC, Lemos VA, Silva LOB, Queiroz AFS, Souza AS, da Silva EGP (2015). Analytical strategies of sample preparation for the determination of mercury in food matrices—a review. Microchem J.

[89] Suvarapu LN, Seo Y-K, Baek S-O (2013). Speciation and determination of mercury by various analytical techniques. Rev Anal Chem.

[90] Corsi P, Venditti I, Battocchio C, Meneghini C, Bruni F, Prosposito P (2019). Designing an optimal ion adsorber at the nanoscale: the unusual nucleation of AgNP/Co2+–Ni2+ binary mixtures. J Phys Chem C.

[91] Elfassy E, Mastai Y, Salomon A (2016). Cysteine sensing by plasmons of silver nanocubes. J Solid State Chem.

[92] Rinaldi F, Del Favero E, Moeller J, Hanieh PN, Passeri D, Rossi M (2019). Hydrophilic silver nanoparticles loaded into niosomes: physical–chemical characterization in view of biological applications. Nanomaterials.

[93] Zając M, Kotyńska J, Zambrowski G, Breczko J, Deptuła P, Cieśluk M (2023). Exposure to polystyrene nanoparticles leads to changes in the zeta potential of bacterial cells. Sci Rep.

[94] Saad PG, Castelino RD, Ravi V, Al-Amri IS, Khan SA (2021). Green synthesis of silver nanoparticles using Omani pomegranate peel extract and two polyphenolic natural products: characterization and comparison of their antioxidant, antibacterial, and cytotoxic activities. Beni-Suef Univ J Basic Appl Sci.

[95] Prosposito P, Burratti L, Venditti I (2020). Silver nanoparticles as colorimetric sensors for water pollutants. Chemosensors..

[96] Sudhakar C, Selvam K, Govarthanan M, Senthilkumar B, Sengottaiyan A, Stalin M (2015). Acorus calamus rhizome extract mediated biosynthesis of silver nanoparticles and their bactericidal activity against human pathogens. J Genet Eng Biotechnol.

[97] Tp A, Rajasekaran A (2012). Method development and validation for the estimation of sildosin in bulk and pharmaceutical dosage forms using UV-VIS spectrophotometry. Asian J Pharm Clin Res.

[98] Rathod BH, Rani SS, Kartheek N, Kumar A (2014). UV spectrophotometric method development and validation for the quantitative estimation of indinavir sulphate in capsules. Int J Pharm Pharmaceut Sci.

[99] Ahmed A-A, Hamzah H, Maaroof M (2018). Analyzing formation of silver nanoparticles from the filamentous fungus *Fusarium oxysporum* and their antimicrobial activity. Turk J Biol.

[100] John MS, Nagoth JA, Ramasamy KP, Mancini A, Giuli G, Natalello A (2020). Synthesis of bioactive silver nanoparticles by a Pseudomonas strain associated with the antarctic psychrophilic protozoon Euplotes focardii. Mar Drugs.

[101] Taha GM, Rashed MN, El-Sadek MSA, Moghazy MAE (2021). Multiferroic BiFeO_3_ dithizone functionalized as optical sensor for detection and determination of some heavy metals in environmental samples. Bull Mater Sci.

[102] Tarnowska M, Krawczyk T (2020). Click chemistry as a tool in biosensing systems for sensitive copper detection. Biosens Bioelectron.

[103] Xie T, Zhong X, Liu Z, Xie C (2020). Silica-anchored cadmium sulfide nanocrystals for the optical detection of copper (II). Microchim Acta.

[104] Liu Z-C, Qi J-W, Hu C, Zhang L, Song W, Liang R-P (2015). Cu nanoclusters-based ratiometric fluorescence probe for ratiometric and visualization detection of copper ions. Anal Chim Acta.

[105] Handayani W, Ningrum AS, Imawan C, editors. The role of pH in synthesis silver nanoparticles using pometia pinnata (matoa) leaves extract as bioreductor2020: IOP Publishing.

[106] Joshi SJ, Geetha S, Al-Mamari S, Al-Azkawi A. Green synthesis of silver nanoparticles using pomegranate peel extracts and its application in photocatalytic degradation of methylene blue. Jundishapur J Nat Pharm Prod. 2018;13(3).

[107] Felimban AI, Alharbi NS, Alsubhi NS (2022). Optimization, characterization, and anticancer potential of silver nanoparticles biosynthesized using *Olea europaea*. Int J Biomater..

[108] Stavinskaya O, Laguta I, Fesenko T, Krumova M (2019). Effect of temperature on green synthesis of silver nanoparticles using *Vitex agnus-castus* extract. Chem J Moldova..

[109] Miri A, Sarani M, Bazaz MR, Darroudi M (2015). Plant-mediated biosynthesis of silver nanoparticles using *Prosopis farcta* extract and its antibacterial properties. Spectrochim Acta Part A Mol Biomol Spectrosc.

[110] Khodadadi S, Mahdinezhad N, Fazeli-Nasab B, Heidari MJ, Fakheri B, Miri A (2021). Investigating the possibility of green synthesis of silver nanoparticles using *Vaccinium arctostaphlyos* extract and evaluating its antibacterial properties. BioMed Res Int.

[111] Lin M, Hu X, Ma Z, Chen L (2012). Functionalized polypyrrole nanotube arrays as electrochemical biosensor for the determination of copper ions. Anal Chim Acta.

[112] Jaffar SS, Saallah S, Misson M, Siddiquee S, Roslan J, Lenggoro W (2023). Green synthesis of flower-like carrageenan-silver nanoparticles and elucidation of its physicochemical and antibacterial properties. Molecules.

[113] Khan S, Almarhoon ZM, Bakht J, Mabkhot YN, Rauf A, Shad AA (2022). Single-step Acer pentapomicum-mediated green synthesis of silver nanoparticles and their potential antimicrobial and antioxidant activities. J Nanomater.

[114] Sithara R, Selvakumar P, Arun C, Anandan S, Sivashanmugam P (2017). Economical synthesis of silver nanoparticles using leaf extract of *Acalypha hispida* and its application in the detection of Mn (II) ions. J Adv Res.

[115] Anis SNS, Liew WC, Marsin AM, Muhamad II, Teh SH, Khudzari AZM (2023). Microwave-assisted green synthesis of silver nanoparticles using pineapple leaves waste. Clean Eng Technol..

[116] Moosa AA, Ridha AM, Al-Kaser M (2015). Process parameters for green synthesis of silver nanoparticles using leaves extract of *Aloe vera* plant. Int J Multi Curr Res.

[117] Ahmed MJ, Murtaza G, Mehmood A, Bhatti TM (2015). Green synthesis of silver nanoparticles using leaves extract of *Skimmia laureola*: characterization and antibacterial activity. Mater Lett.

[118] Krithiga N, Rajalakshmi A, Jayachitra A (2015). Green synthesis of silver nanoparticles using leaf extracts of *Clitoria ternatea* and *Solanum nigrum* and study of its antibacterial effect against common nosocomial pathogens. J Nanosci.

[119] Taufiq M, Eden WT, Sumarni W, Alauhdin M, editors. Colorimetric detection of metal ions using green-synthesized silver nanoparticles. 2012 2021: IOP Publishing.

[120] Kazemi Z, Marahel F, Hamoule T, Mombeni Goodajdar B (2021). Removal of Ni (II) and Co (II) ions from aqueous solutions utilizing Origanum majorana-capped silver nanoparticles. Desalin Water Treat.

[121] Kim H-T, Lee TG (2017). A simultaneous stabilization and solidification of the top five most toxic heavy metals (Hg, Pb, As, Cr, and Cd). Chemosphere.

[122] Kim E, Horckmans L, Spooren J, Vrancken KC, Quaghebeur M, Broos K (2017). Selective leaching of Pb, Cu, Ni and Zn from secondary lead smelting residues. Hydrometallurgy.

[123] Zhang B, Huo X, Xu L, Cheng Z, Cong X, Lu X (2017). Elevated lead levels from e-waste exposure are linked to decreased olfactory memory in children. Environ Pollut.

[124] Wei S, Wang Y, Tang Z, Hu J, Su R, Lin J (2020). A size-controlled green synthesis of silver nanoparticles by using the berry extract of Sea Buckthorn and their biological activities. New J Chem.

[125] Pan N, Maji TK, Sayantika Bandyopadhyay PB, Chatterjee A, Mala Mitra AC, Pal SK (2022). A combined spectroscopic and theoretical analysis of plasmonic silver nanoparticles sensor towards detailed microscopic understanding of heavy metal detection. Plasmonics.

[126] Ahmed F, Kabir H, Xiong H (2020). Dual colorimetric sensor for Hg^2+^/Pb^2+^ and an efficient catalyst based on silver nanoparticles mediating by the root extract of *Bistorta amplexicaulis*. Front Chem.

[127] Yang Q, Tan Q, Zhou K, Xu K, Hou X (2005). Direct detection of mercury in vapor and aerosol from chemical atomization and nebulization at ambient temperature: exploiting the flame atomic absorption spectrometer. J Anal At Spectrom.

[128] Khusnuriyalova AF, Caporali M, Hey-Hawkins E, Sinyashin OG, Yakhvarov DG (2021). Preparation of cobalt nanoparticles. Eur J Inorg Chem.

[129] Saha P, Billah MM, Islam ABMN, Habib MA, Mahiuddin M (2023). Green synthesized silver nanoparticles: a potential antibacterial agent, antioxidant, and colorimetric nanoprobe for the detection of Hg2+ ions. Glob Chall..

[130] Sanjeevappa HK, Nilogal P, Rayaraddy R, Martis LJ, Osman SM, Badiadka N (2022). Biosynthesized unmodified silver nanoparticles: a colorimetric optical sensor for detection of Hg2+ ions in aqueous solution. Results Chem..

[131] Sharaf Zeebaree SY, Haji OI, Zeebaree AYS, Hussein DA, Hanna EH (2022). Rapid detection of mercury ions using sustainable natural gum-based silver nanoparticles. Catalysts.

[132] Guo X, Huang J, Wei Y, Zeng Q, Wang L (2020). Fast and selective detection of mercury ions in environmental water by paper-based fluorescent sensor using boronic acid functionalized MoS2 quantum dots. J Hazard Mater.

[133] Yadav R, Berlina AN, Zherdev AV, Gaur MS, Dzantiev BB (2020). Rapid and selective electrochemical detection of pb 2+ ions using aptamer-conjugated alloy nanoparticles. SN Appl Sci.

[134] Azimi H, Ahmadi SH, Manafi M, Mousavi SHH, Najafi M (2020). Development of an analytical method for the determination of lead based on local surface plasmon resonance of silver nanoparticles. Quim Nova.

[135] Khan NA, Niaz A, Zaman MI, Khan FA, Tariq M (2018). Sensitive and selective colorimetric detection of Pb2+ by silver nanoparticles synthesized from *Aconitum violaceum* plant leaf extract. Mater Res Bull.

[136] Wang H-B, Ma L-H, Fang B-Y, Zhao Y-D, Hu X-B (2018). Graphene oxide-assisted Au nanoparticle strip biosensor based on GR-5 DNAzyme for rapid lead ion detection. Colloids Surf, B.

[137] Rajar K, Alveroglu E, Caglar M, Caglar Y (2021). Highly selective colorimetric onsite sensor for Co2+ ion detection by povidone capped silver nanoparticles. Mater Chem Phys.

[138] Yao Y, Tian D, Li H (2010). Cooperative binding of bifunctionalized and click-synthesized silver nanoparticles for colorimetric Co2+ sensing. ACS Appl Mater Interfaces.

[139] Sharma S, Jaiswal A, Uttam KN (2021). Synthesis of sensitive and robust lignin capped silver nanoparticles for the determination of Cobalt (II), Chromium (III), and Manganese (II) ions by colorimetry and Manganese (II) ions by surface-enhanced Raman scattering (SERS) in aqueous media. Anal Lett.

[140] Zafer M, Keskin CS, Özdemir A (2020). Highly sensitive determination of Co (II) ions in solutions by using modified silver nanoparticles. Spectrochimica Acta Part A: Mol Biomol Spectrosc..

[141] Wang Y, Dong X, Zhao L, Xue Y, Zhao X, Li Q (2020). Facile and green fabrication of carrageenan-silver nanoparticles for colorimetric determination of Cu2+ and S2−. Nanomaterials.

[142] Ghodake GS, Shinde SK, Saratale RG, Kadam AA, Saratale GD, Syed A (2018). Colorimetric detection of Cu2+ based on the formation of peptide–copper complexes on silver nanoparticle surfaces. Beilstein J Nanotechnol.

[143] Zhou Y, Zhao H, He Y, Ding N, Cao Q (2011). Colorimetric detection of Cu2+ using 4-mercaptobenzoic acid modified silver nanoparticles. Colloids Surf A: Physicochem Eng Aspects.

[144] Wu G, Dong C, Li Y, Wang Z, Gao Y, Shen Z (2015). A novel AgNPs-based colorimetric sensor for rapid detection of Cu 2+ or Mn 2+ via pH control. RSC Adv.

[145] Vopálenská I, Váchová L, Palková Z (2015). New biosensor for detection of copper ions in water based on immobilized genetically modified yeast cells. Biosens Bioelectron.

